# Reconstruction of full-length circular RNAs enables isoform-level quantification

**DOI:** 10.1186/s13073-019-0614-1

**Published:** 2019-01-19

**Authors:** Yi Zheng, Peifeng Ji, Shuai Chen, Lingling Hou, Fangqing Zhao

**Affiliations:** 1 Computational Genomics Lab, Beijing Institutes of Life Science, Chinese Academy of Sciences, Beijing, 100101 China; 2 University of Chinese Academy of Sciences, Beijing, 100049 China; 3 Center for Excellence in Animal Evolution and Genetics, Chinese Academy of Sciences, Kunming, 650223 China

**Keywords:** Alternative splicing, Circular RNA (circRNA), Transcript reconstruction, Isoform quantification

## Abstract

Currently, circRNA studies are shifting from the identification of circular transcripts to understanding their biological functions. However, such endeavors have been limited by large-scale determination of their full-length sequences and also by the inability of accurate quantification at the isoform level. Here, we propose a new feature, reverse overlap (RO), for circRNA detection, which outperforms back-splice junction (BSJ)-based methods in identifying low-abundance circRNAs. By combining RO and BSJ features, we present a novel approach for effective reconstruction of full-length circRNAs and isoform-level quantification from the transcriptome. We systematically compared the difference between the BSJ-level and isoform-level differential expression analyses using human liver tumor and normal tissues and highlight the necessity of deepening circRNA studies to the isoform-level resolution. The CIRI-full software can be accessed at https://sourceforge.net/projects/ciri.

**Electronic supplementary material:**

The online version of this article (10.1186/s13073-019-0614-1) contains supplementary material, which is available to authorized users.

## Background

Circular RNA (circRNA) is a type of RNA molecules in which both ends are covalently linked. Advances in deep sequencing and identification algorithms have resulted in a huge number of circRNAs from fly to human [1–6]. Most recently, new subclasses of circRNAs, including non-exonic circRNAs [7, 8] and exon-intron circRNAs [9], have been explored. Subsequent studies unveiled the ubiquity of alternative splicing (AS) events within circRNAs and revealed a profound difference in the expression of circRNAs and mRNAs [10]. Profiting from these identified circRNAs, most recent studies have shifted to the efforts of revealing the biological functions of circRNAs. As a heterogeneous class, circRNAs may participate in various aspects of biological processes. In addition to the well-studied function of microRNA sponges [3, 4], studies have illustrated that these circular transcripts may be involved in gene regulation [11], development [12], innate immune response [13], and diseases [14–21]. Recent efforts have shown that N^6^-methyladenosine (m^6^A) promotes the efficient initiation of protein translation from circRNAs [22]. Subsequently, Zhou et al. demonstrated the prevalence of m^6^A in circRNAs [23]. Collectively, these intriguing findings illustrate the complexity of circRNA functions and show that our understanding of how circRNAs participate in biological processes is still rudimentary.

Evolutionary analyses of gene sequences and expression patterns have provided essential insights into gene functional study. Increasing attention has also been paid to evolutionary analysis of circRNAs among different species. Rybak-Wolf et al. systematically compiled a catalog of neuronal human and mouse circRNAs and found that these circRNAs were preferentially enriched in mammalian brain and that the same circRNAs were often expressed in both species and were well conserved in sequence [24]. As the first systematic analysis of circRNAs in mammalian brains, this work represents an important step toward further elaboration of circRNA functions. However, owing to the lack of full-length circRNAs, sequence conservation comparison is restricted to flanking introns and coding DNA sequences (CDSs). Considering the prevalence of circRNA isoforms generated by combinations of internal components within back-splice junctions (BSJs), current cross-species conservation analyses based on partial sequences may lead to biased estimation of circRNA conservation with respect to expression pattern and sequence composition. Moreover, using BSJs to represent a collection of different circRNA isoforms hampers our understanding of specific circRNA functions and makes it difficult to achieve further evolutionary insights into circRNAs among species. A fully automated method that can identify large-scale full-length circRNAs from RNA-seq data has yet to be developed.

A number of efforts have recently been made to explore the internal landscape of circRNAs. Some studies utilized a straightforward strategy that simply combined all known mRNA exons in a sequential order as putative full-length circRNA [2, 25]. This method, however, relies on an unsupported assumption that circular and linear transcripts share the same composition and thus may lead to misunderstanding in downstream analyses. Other methods, such as CIRI-AS [10], CIRCexplorer2 [26], and FUCHS [27], involve identifying the internal components of the BSJ. CIRI-AS employs a spliced junction signature-based algorithm and enables, for the first time, high-throughput detection of internal components of circRNAs based on short-read sequencing. Similar to CIRI-AS, FUCHS predicts circRNA internal sequences by extracting the mapping results of BSJ reads. In an alternative manner, CIRCexplorer2 detects alternative splicing events through comparison analysis between poly(A)+ and poly(A)− RNA-seq data sets. However, without considering combinations of these components, whole-sequence prediction of circular isoforms with complicated AS events is still beyond the reach of these methods. Most recently, Ye et al. employed a strategy similar to CIRI-AS to assemble full-length sequences of circRNAs using BSJ read pairs, but this approach still faces an inherent challenge in that only a small fraction of circRNAs can be identified by assembling BSJ reads [28]. As a result, without whole-sequence of circRNAs, the reconstruction of circular isoforms within a certain BSJ extends far beyond the scope of current analysis and accurate quantification of circRNAs at the isoform level remains an insurmountable obstacle. Therefore, the inability of reconstructing full-length circRNAs and quantifying circular isoforms places limitations on the discovery of previous unknown biological phenomena, which may restrict our ability to understand the diversity and expression patterns of circRNA isoforms.

To address this challenge, we present a new feature, reverse overlap (RO), for full-length circRNA reconstruction and isoform-level quantification. RO is especially suitable for identifying low-abundance circRNAs that are difficult to identify using the BSJ feature. Considering that a vast majority of circRNAs in various transcriptomes are in low expression levels, the detection of such low-abundance transcripts is extremely important in circRNA studies. Moreover, we develop an accurate, high-throughput approach (CIRI-full) that uses both BSJ and RO features to reconstruct full-length circRNAs and circular isoforms within them from RNA-seq data sets. Several recent independent studies demonstrated that CIRI2 exhibited remarkably balanced sensitivity, reliability, running time, and RAM usage on circRNA detection [29–32]. Most recently, Thomas B. Hansen further systematically compared 11 circRNA detection algorithms and found that CIRI2 was one of the best algorithms for circRNA identification and performed comparably to annotation-based algorithms [33]. In CIRI-full, CIRI2 is employed to detect cirexons (circRNA’s exon) and to determine the boundaries of circRNAs. The RO feature, which is deduced from reversely overlapped paired-end reads, is used to explore the detailed cirexon landscape within boundary sites and to assemble into full-length sequence. Based on the assembled full-length circRNAs, a forward splice graph (FSG)-based algorithm is employed to reconstruct all full-length isoforms within them and to determine their abundances. Compared with previous methods, CIRI-full is not only efficient at determining complete sequences of circRNAs, but, more importantly, enables the analysis of circRNAs at the isoform level. We applied CIRI-full to survey circRNA expression patterns in samples from the brains of six vertebrates and also explored circRNA expression divergence between tumor and normal tissues at both BSJ- and isoform-level resolution, which uncovered distinct expression patterns between circRNAs and their isoforms. This study presents an important approach to assemble and quantify circRNAs and will greatly improve our understanding of their biogenesis and functions.

## Methods

### Overview of CIRI-full

The CIRI-full algorithm is a four-step process that includes RO read detection and verification, BSJ and cirexon detection, combined assembly of both RO and BSJ reads, and isoform reconstruction and quantification. RO read detection and verification is designed to detect RO reads from paired-end reads based on their 5′ reverse overlaps and to rule out linear transcripts and false positives resulting from lariat structures using sophisticated post-alignment filters. An RO-merged read is identified as full-length circRNA if the genomic alignments of both its ends either have overlaps or are located in the same cirexon. The BSJ and cirexon detection step was developed to detect BSJs and cirexons and to identify single-splice events. If the BSJ read pairs are exclusively located on all the cirexons within the BSJ, the complete sequence of this circRNA can be reconstructed using these cirexons. RNA-seq reads are processed separately in the first two steps, resulting in the identification of a number of full-length circRNAs in addition to BSJs and RO-merged reads that are not sufficient for reconstructing full-length circRNA independently. Therefore, these unused but informative RO-merged reads and BSJ reads are integrated in make a combined assembly. Finally, for a given BSJ, all forward splice junctions are recognized and quantified, and then an adapted FSG [34–36] is built to estimate the isoform expression abundance.

### RO read detection and verification

During the reverse transcription step of library preparation, transcription begins at the primers and walks along the RNA template. Owing to the unique structure of circRNAs, these circular transcripts will be repeatedly reverse-transcribed. When sequencing these reverse-transcribed cDNAs, peculiar reverse overlap features on the read pair will be observed on the pair-end reads if the library insert length is greater than the length of the circRNA. Specifically, the 5′- or 3′-ends on both paired reads are reversely overlapped with each other, which can be used as an indicator of circRNA. Moreover, it should be noted that the presence of 3′-RO on both reads indicates that the complete sequence of the circRNA has been read, and thus, its whole sequence can be reconstructed.

The 5′ RO reads are identified based on the following strategy. For each read pair, the first 10 bp at the 5′-end of one read is divided into three subsequences with a window size of 8 bp and a step size of 1 bp. These subsequences are then used as seeds to search for matches at the 5′-end of the other read. Once all these seeds have matches (match base pair ≥ 7 bp) on the other read, the sequence with location ranging from the 5′ terminus to the matches on this read is extracted and aligned to the counterpart read. Both members of this pair of reads are taken as candidate 5′ RO reads if the alignment satisfies two criteria: (i) aligned length ≥ 13 bp and (ii) nucleotide acid identity ≥ 95%. Subsequently, these two reads are merged into a long read based on the alignment, and the long read is treated as a candidate RO-merged read for further validation.

During the library preparation step, the size of fragments is not strictly the same as the library insert length but varies around the insert length. Moreover, if the fragment size is shorter than the sequenced read length, this will yield partial adapter sequences attached to the 3′-ends of paired-end reads. The presence of this type of read, which indeed originates from linear transcripts, can lead to false-positive 5′ RO features when performing candidate RO read detection. To rule out such false-positive RO reads, a mapping-based filtration strategy is employed. First, the precise location of the candidate RO-merged read is determined. Specifically, the candidate RO-merged read is aligned to the reference genome using BWA-MEM (-T 19, minimum score to output), which outputs split alignments of the read on the genome. The alignments are then collected and sorted according to their alignment lengths. The longest alignment with mapping quality greater than 15 is used as an anchor alignment, and the accumulated mapping length within a 100-Kbp interval on both sides of this anchor alignment is calculated. If the summed length exceeds half of the read length, this candidate RO-merged read is reserved for further analysis. Otherwise, it is discarded. Finally, the reserved candidate RO-merged reads are remapped using a local realignment strategy to accurately determine their locations. Briefly, highly reliable mapping fragments are determined using the BSJ position or, when no BSJ position is available, the anchor position. Then, the precise locations of the unmapped or abnormal mapped fragments are obtained using dynamic programming. After determining the locations of the candidate RO-merged reads, linear transcript-derived reads are identified and ruled out if they satisfy two criteria [1]: the reads contain no BSJ and [2] the subsequences with the same length on both ends of the read have no hit around the anchor alignment.

Because BWA-MEM was originally designed for mapping DNA sequencing reads, this tool does not consider GT/AG splicing signals and fails to obtain the accurate boundaries of split alignments in which the mapping position may deviate by a few base pairs from the true boundary position. Moreover, use of BWA-MEM may also lead to the inclusion of lariat structures in the candidate RO reads. To justify the boundaries and remove the lariat structures, the alignments for each candidate RO-merged read are revisited by checking for the presence of a GT/AG splicing signal. For each read, the GT/AG splicing site of the aligned fragments is first checked, and the read is filtered if any aligned fragment does not contain a GT/AG splicing site. Then, the GT/AG splicing sites on the remaining candidate RO reads are justified. Next, the alignments of two 5-bp subsequences from both sides of the junction site on each read are extracted, and whether these alignments have mapping gaps or mismatches is determined. If there is no gap or mismatch in these alignments, this read is taken as a highly reliable RO-merged read. Otherwise, it is discarded.

### BSJ and cirexon detection

BSJs in the RNA-seq reads are detected using CIRI2 [30], and single-splice events within these BSJs are inferred from CIRI-AS (parameter -d yes) [10]. Within each BSJ, all cirexons inferred from the single-splice events are collected, sorted, and recorded.

### Combined assembly of RO and BSJ reads

After identifying RO-merged reads and BSJ boundaries, full-length circRNAs are identified separately using these two types of information. Specifically, an RO-merged read is identified as a full-length circRNA if the genomic alignments of its both ends either have overlaps (3′ RO) or are located on the same cirexon. Otherwise, the RO-merged reads are reserved for further combined assembly. For each BSJ, the alignments of all the paired-end BSJ reads are collected; if the BSJ reads are all exclusively located on cirexons, the complete sequence of this circRNA is reconstructed by linearly connecting the cirexons. Otherwise, the BSJ and the cirexons within them are recorded for further combined assembly.

The BSJ feature provides an efficient method of detecting the existence of circRNAs but cannot be used to identify the internal composition of the circRNA. The RO feature greatly facilitates determination of the internal compositions of circRNAs but sometimes may fail to cover the BSJ site owing to the limitation of read length. Hence, CIRI-full employs a combined strategy that uses the advantages of both BSJ and RO features to reconstruct a more comprehensive full-length circRNA repository. By utilizing the unused RO-merged reads and BSJ reads, full-length circRNAs are reconstructed using the following process. The RO-merged reads and BSJ reads are sorted and clustered according to the BSJ. If both types of read are observed within a BSJ, the reads are used to reconstruct full-length circRNAs. For each circRNA, the RO-merged reads are used to determine additional cirexons that were not identified by the BSJ reads, and the alignments of all the BSJ read pairs are re-checked. If these reads are all exclusively located on cirexons, the complete sequence is reconstructed by linearly connecting the cirexons. Otherwise, the identified cirexons within the BSJ are outputted and marked as a partially reconstructed circRNA.

### Quantifying the expression of circRNAs at the isoform level

After obtaining the reconstructed circRNAs, CIRI-full builds a forward splice graph (FSG) using all BSJ reads and RO-merged reads within each BSJ. The nodes in the FSG represent cirexons, and the edges represent their connections (i.e., forward splice junctions between cirexons). Theoretically, the FSG covers all possible circular isoforms that are consistent to the mapped reads within the BSJ, and the traversing path from the start cirexon to the end cirexon represents a candidate splicing isoform. It should be noted that the FSG is a closed circuit owing to the nature of circRNAs. Then, an adapted depth-first search (DFS) algorithm is performed to exhaustively decompose the FSG graph into paths. Specifically, the DFS algorithm starts iteratively at each node and stops at the breakpoints (without continuous splicing events) or the start cirexon. After obtaining these paths, short paths are merged into longer paths and redundant paths are then filtered. To avoid a large number of false-positive paths which will significantly affect the efficiency of later iteration steps, CIRI-full screens out a certain number (by default, set to 10) of paths. In detail, the edges on the FSG graph are categorized into four types: (i) BSJ, (ii) phasing FSJ, where the splicing event is exclusively occupied by only one circular isoform, (iii) co-occurred FSJs, where the number of splicing events is supported by the same RO read, and (iv) the remaining FSJs. Paths containing phasing FSJ and co-occurred FSJs will give the top priority for screening, and the corresponding paths are referred to as phased isoforms, because these circular isoforms undoubtedly exist. Regarding the paths that contain the remaining FSJs, they are sorted using the node sequencing depth and the paths with high-sequencing depth are retained to fill up the threshold (by default, set to 10). The resulting outputted paths are referred to as candidate isoforms.

Next, the following steps are to determine the relative abundance for each path. In detail, a Monte Carlo simulation method is used to simulate the BSJ-reads distribution on each path based on the insert length distribution of the RNA-seq library, which is inferred from mapping distance of paired-end reads. According to the distribution of simulated reads, the abundance of nodes and edges (splicing events) on each path can be calculated in the latter steps. To quantify the relative abundance of each path, an approximate exhaustive search algorithm is proposed. Specifically, this approach starts by assigning a random putative abundance (positive integral value) to each path, where the summed abundance for all paths should be equal to the total number of BSJ reads. Based on the assigned putative abundance and the distribution of simulated BSJ reads, the putative abundances of nodes and edges on each path are computed. Based on the resulting abundance of nodes and edges, accumulated putative abundance of nodes and edges are calculated. Then, the distance between putative and real abundance (inferred from mapped BSJ reads) of nodes and edges is calculated and recorded. The putative abundances of paths are adapted to real abundance of nodes and edges. Next, it iterates through the following steps. The putative abundances of nodes and edges on each path are re-calculated. Accumulated putative abundance of nodes and edges are computed. The distance between putative and real abundance (inferred from mapped BSJ reads) of nodes and edges is calculated and compared with pre-recorded distance. If this distance is larger than the pre-recorded distance, it means that the path abundance adaption process goes wrong and new path abundance adaption process is performed. Otherwise, the distance is recorded, new iteration starts. This iteration process stops when the distance converges. By this method, we can obtain the relative abundance for circular isoforms of a certain circRNA.

### Simulated data sets for circRNA identification and reconstruction

Simulated data sets were generated by a CIRI simulator [8]. This tool can simulate RNA-seq data with given references and annotations. Parameters such as read length, coverage, sequencing error rate, and insert size can be customized. Insert length distribution L follows a normal distribution (μ, σ^2^). For each pair of reads, the insert length size is generated using two independent uniformly distributed random numbers *x*_1_, *x*_2_ (0 < *x*_1_, *x*_2_ < 1), as follows:

$$ \mathrm{L}=\sqrt{2\times \ln \left({x}_1\right)}\times \cos \left(2\pi {x}_2\right)\ast \sigma +\mu $$

To make the simulated circRNA more closely resemble real data, the length distribution and expression distribution of the circRNAs were also considered. We first applied CIRI-AS to RNA-seq data from the HeLa cell line (SRA accession number: SRR3476956) to predict the size of identified circRNAs by summing all cirexon lengths with supporting BSJ reads > = 5.

We also adjusted the sequencing depth of the circRNAs in the simulated data according to the real data. For inputting parameter sequencing depth D, the coverage of a particular circular transcript was generated using a uniformly distributed random number *x* (0 < *x* < 1) as follows:

$$ \mathrm{Coverage}=\left(\left(\mathrm{D}-0.5\right)\times 3+1\right)/\left(1-\sqrt{x}\right) $$

To validate the robustness of the RO feature, two groups of simulated paired-end transcriptomic data sets were used to test the performance of the RO detection method. The first group was designed to test the performance of the RO feature under different sequencing depths. This group consisted of four circular transcript sequencing datasets with different average depths (2×, 5×, 10×, and 15×). The read length was set to 200 bp, and the insert length distribution was set to *μ* = 350 bp, *σ* = 200 bp. The second group was constructed to test the performance of the RO feature under different sequencing lengths. This group contained four sets of circular transcript sequencing data with different read lengths (75 bp, 100 bp, 150 bp, and 200 bp). The average depth of these four datasets was 10×, and the insert length followed the same distribution as that of the former group. Furthermore, BSJ-based circRNA detection tools, including CIRI2 [30], find_circ [4], CIRCexplorer2 [25], and KINFE [37], were used for performance comparison.

### Simulated data sets for circRNA isoform quantification

To compare the performance of circRNA isoform quantification between CIRI-full and CIRI-AS, we simulated circRNA-containing RNA-seq data sets, where two different isoforms were added for each circRNA by simulating additional exon skipping event. Note that the number of isoforms for each circRNA was set to two, because only in this situation, CIRI-AS can estimate the relative abundance of the two isoforms within a certain circRNA using the PSI values. Consequently, two data sets were simulated. The first one simulated transcripts with different sequencing depth (25×, 50×, 100×, and 150×, respectively) and uniform read length of 150 bp with insert length of 350 ± 200 bp. The second one simulated transcripts with different read length (100, 150, 200, 250, and 300 bp, respectively), insert length of 350 ± 200 bp and sequencing depth of 75×.

To further evaluate the sensitivity and accuracy of CIRI-full on circRNA isoform detection and quantification, we also simulated circRNA-containing RNA-seq data sets with three isoforms for each circRNA. The transcript sequencing depth was set to 50× with sequencing length of 150 bp and insert length of 350 ± 200 bp.

### Generation of HeLa cell RNA-seq data

Total RNA was isolated using TRIZOL (Invitrogen) from HeLa cells grown in standard medium under standard conditions. The RNA was divided into three samples containing equal amounts of RNA. The quality of these samples was manually controlled to produce different RIN values. Specifically, the RIN value of the low-quality RNA sample was 5 and that of the two high-quality samples was 10. Next, a RiboMinus kit (Invitrogen, Carlsbad, CA, USA) was utilized to deplete ribosomal RNA in these samples. The resulting RNA was incubated at 37 °C and treated with 10 U μg^− 1^ RNase R (Epicenter, Madison, WI, USA). One of the high-RIN samples and the low-RIN sample were used separately as templates for cDNA libraries following the TruSeq protocol (Illumina, San Diego, CA, USA); the other high-RIN sample was used to construct a sequencing library using the same protocol but without the fragmentation step. Fragments with a broad range of fragment size (300–800 bp) were selected for library construction. The three libraries (low RIN/fragmented, high RIN/fragmented, high RIN/unfragmented) were sequenced on the Illumina HiSeq 2500 platform of the Research Facility Center at the Beijing Institutes of Life Science, CAS, with a read length of 250 bp. The sequencing data sets have been deposited to SRA with the following project ID (PRJNA475651) [38].

### Generation of whole brain RNA-seq data

Human, macaque, and rabbit whole brain RNA samples were purchased from Zyagen (San Diego, CA, USA). Mouse, rat, and chicken whole brain tissues were obtained from the Research Facility Center at the Beijing Institutes of Life Science, CAS. RNA samples were isolated using TRIZOL (Invitrogen). For each species, three types of cDNA library were prepared. Specifically, a Ribo-/RNase R library was constructed using RNA samples that had been treated with the RiboMinus kit (Invitrogen) and then incubated at 37 °C with 10 U μg^−1^ RNase R, a Ribo-/cDNA library was constructed using RNA samples that were only treated with the RiboMinus kit, and a poly-A library was prepared according to the TruSeq v2 guide. Poly-A and Ribo-RNA samples were used as templates for cDNA libraries according to the TruSeq protocol (Illumina); Ribo-/RNase R-treated samples used the same protocol but without fragmentation. These libraries were sequenced on the Illumina HiSeq 2500 platform of the Research Facility Center at Beijing Institutes of Life Science, CAS. PolyA+ and Ribo-/RNase R libraries were sequenced with paired-end 250-bp reads, and Ribo-libraries were sequenced with paired-end 150-bp reads. The sequencing data sets have been deposited to SRA with the following project ID (PRJNA475651) [38].

### HeLa cell and brain RNA-seq data processing

CircRNAs from three HeLa cell RNA-seq data sets were identified using CIRI-full with default parameters. The RNA-seq data sets of brain samples that had undergone RiboMinus/RNase R treatment from six species were processed using CIRI-full. To normalize the number of resulting circRNAs, circRNAs with no RO read support and those with BSJ read support ≤ 5 were filtered in human, mouse, rat, and rabbit according to the total BSJ number. Brain data sets from RiboMinus libraries were processed by CIRI2 with default parameters, and expression level was normalized to data set size. Poly-A-selected RNA-seq data sets of brain samples of the six vertebrates were analyzed using Hisat2 [39] and StringTie [40, 41] with default parameters.

### Experimental validation

To validate the predictions made by the RO method, outward-facing primer sets were designed to amplify 21 circRNAs (15 for BSJ validation and 6 for full-length circRNA validation). PCRs were performed using 35 cycles, and the sequences of the PCR products were determined via Sanger sequencing. Among 21 validated circRNAs, all BSJs and FSJs of six circRNAs were supported by Sanger sequencing, indicating the accuracy of the reconstructed full-length sequences of these circRNAs. The remaining 15 circRNAs were also validated by the confirmed BSJ sites using Sanger sequencing.

To verify predicted circRNA isoforms and their relative abundances, outward-facing primers were designed to quantify the expression of 17 isoforms within 8 circRNAs. Specifically, HeLa cells were grown in standard media and conditions. Total RNA was isolated using TRIZOL and converted to cDNA using random hexameters primers within the FastKing RT Kit (TIANGEN). Resulting cDNA was used as templates and real-time qPCR was performed using primer pairs specific for circRNA isoforms and two negative controls (GAPDH and b-actin) with SYBR FAST qPCR Kits (Kapa Biosystems). The reaction volume was 20 μl, which contained 1 μl of serial diluted cDNA, 10 μl of qPCR SYBR Green Master Mix, 0.5 μl each of forward and reverse primers, and 8 μl of water. Thermal cycling was carried out on StepOnePlus (Applied Biosystems) using the following conditions: 95 °C for 5 min and followed by 40 cycles of 95 °C for 10 s and 60 °C for 30 s. Fluorescent signals were detected at the step of annealing/extension (60 °C).

### Differential expression analysis of circRNAs and their isoforms in HCC patients

RiboMinus treated RNA-seq data sets of tumor (SRX1558046-SRX1558064) and normal liver samples (SRX1558026-SRX1558045) from 20 HCC patients generated in a previous study [42] were downloaded from the NCBI SRA database. Full-length circRNAs and their isoforms were obtained by running CIRI-full on these data sets using default parameters. The statistical significances of differentially expressed circRNAs between normal and tumor samples were calculated using Mann–Whitney *U* test.

## Results

Currently available approaches on circRNA identification are exclusively based on the detection of back-splice junctions (BSJs). In this study, we propose a new feature, reverse overlap (RO), for full-length circRNA detection, which outperforms previous BSJ-based methods in detecting circRNAs, even for highly degraded RNA samples. We further develop an accurate and high-throughput approach, CIRI-full, that uses both BSJ and RO features to reconstruct full-length circRNAs and their isoforms from RNA-seq data sets.

### The CIRI-full approach

**Fig. 1:**
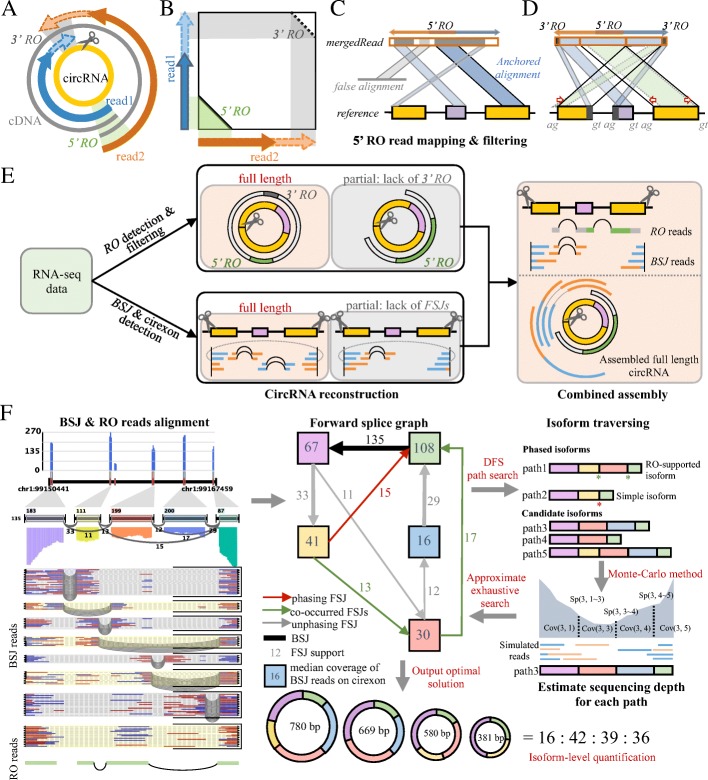
Workflow of reverse overlap detection and full-length circular RNA reconstruction. RO, reverse overlap; BSJ, back-spliced junction; FSJ, forward-spliced junction. **a** RO is an overlapped region in amplified circular transcripts in which the 5′- or 3′- ends of paired reads are reversely overlapped with each other. The presence of a 5’ RO indicates that the paired reads are derived from a circular transcript. The presence of both 5′ and 3′ ROs indicates that a full-length circular transcript can be generated by merging the 5′ and 3′ overlapped sequences of the read. **b** Alignment of a read pair with 5′ RO and/or 3′ RO. **c**, **d** Candidate RO-merged reads are mapped to the reference genome to accurately determine the locations of the reads and to rule out contamination. The longest alignment is chosen as an anchor for determining the location of the reads (**c**). Unmapped and abnormally mapped fragments in the candidate RO-merged reads are realigned to the reference genome based on the location of the anchored alignment; the alignment boundaries are then adjusted based on the GT/AG splicing signal (**d**). **e** Workflow of full-length circRNA reconstruction. ROs, BSJs and cirexons are first detected from RNA-seq data. Full-length circRNAs can be reconstructed when both 5′ and 3′ RO are present or when the circRNAs are completely covered by BSJ reads. For circRNAs lacking 3′ RO or FSJs, a combined assembly is performed to integrate the 5′ RO reads and the BSJ reads. **f** Isoform-level quantification of circRNAs. The BSJ and RO-merged reads are aligned to the reference genome. A forward splice graph (FSG) that records the splicing and coverage information is built based on the alignments. Next, the resulting FSC is dissected into paths that represent putative circular isoforms of the circRNA (right panel). Paths that contain phasing FSJ, where the splicing event is exclusively occupied by only one circular isoform, or co-occurred FSJs, where the number of splicing events is supported by the same RO read, are classified as phased isoforms. The read coverage profile of each path is modeled by a Monte Carlo simulation (right middle panel). Expressed circular isoforms are dissected and quantified by employing an approximate exhaustive search algorithm (bottom)

The RO identification algorithm (Additional file 1: Figure S1) is performed based on region in amplified circular transcripts in which the 5′- and 3′- ends of paired reads are reversely overlapped with each other (Fig. 1a, b). It should be noted that 3′- end RO (3′ RO) will occur if the circRNA is completely covered by paired-end reads; in this case, the entire sequence of the circRNA can be reconstructed (Fig. 1a). However, the presence of RO features is dependent on library fragment size, cDNA amplification, and read length (Additional file 1: Figure S2). This method involves two steps, RO detection and RO verification. The former is designed to detect candidate RO reads. Specifically, for each read pair, the 5′- end subsequences of both reads are extracted and aligned using a seed-matching strategy. The read pairs for which 5′- end subsequence alignment passes the length and identity thresholds are merged into a long sequence according to the alignment and taken as candidate RO-merged reads. Owing to contamination with lariat structures and linear transcript reads that have partial sequences attached to both ends, a significant number of candidate RO-merged reads are false positives. Next, a mapping-strategy-based RO verification step is utilized to screen out authentic RO-merged reads. In this step, candidate RO-merged reads are mapped to the reference genome, resulting in several split alignments. To accurately determine the location of candidate RO-merged reads, the longest alignment for each read is employed as an anchor alignment, and the accumulated mapping length within a given interval on both sides of the anchor is calculated. If the summed length exceeds half of the read length, the read is reserved and remapped using a local realignment strategy. Abnormally mapped and unmapped regions for each read are remapped using local realignment; then, the boundaries of the mapping junctions are adjusted according to the GT/AG splicing signal. Moreover, candidate RO-merged reads derived from linear transcripts and lariat structures are also ruled out in this process. Finally, the locations of both ends of the remaining RO-merged reads are checked and recorded for further analyses (Fig. 1c, d and Additional file 1**:** Figure S3).

The CIRI-full pipeline involves four different steps, RO detection and reconstruction, BSJ and cirexon detection, combined assembly of RO and BSJ reads (Fig. 1e) and circular isoform detection and quantification (Fig. 1f). Several recent studies demonstrated that CIRI2 exhibited remarkably balanced sensitivity, reliability, running time, and RAM usage on circRNA detection [29–32] and performed comparably to annotation-based algorithms [33], and thus, CIRI2 was employed to detect BSJ reads in this pipeline. RNA-seq reads are processed separately in the first two steps, thus yielding a number of full-length circRNAs, as well as BSJ and RO-merged reads, which are not sufficient for reconstructing full-length circRNA independently. Therefore, these unused but informative reads are integrated in the next step to generate a combined assembly. Based on the identified full-length circRNAs, circular isoforms within the BSJs of a circRNA are then detected and quantified by employing statistic-based models. In the first step, an RO-merged read is identified as full-length circRNA if the genome alignments of both of its ends satisfy one of two criteria [1]: they have overlap on the genome (Additional file 1: Figure S4A) or [2] they do not have overlap but locate on the same cirexon (Additional file 1: Figure S4B). In the second step, CIRI-AS is employed to detect BSJ and cirexons of circRNAs. Then, for each circRNA, the locations of BSJ read pairs within the BSJ are checked; if all the reads are exclusively located on the cirexons, the complete sequence of this circRNA is assembled by linearly connecting the cirexons (Additional file 1: Figure S4C). In the third step, incomplete information from the first two steps is clustered according to the BSJ, and the RO reads and cirexons for each BSJ are combined to complement each other to reconstruct full-length circRNAs (Additional file 1: Figure S4D). However, under certain conditions, full-length circRNAs cannot be reconstructed; these may include long circRNAs whose length is twofold greater than the library size (Additional file 1: Figure S5D) and circRNAs with incomplete cirexons due to low expression levels (Additional file 1: Figure S5A–C). Finally, a forward splice graph (FSG) is constructed using the BSJ and RO-merged reads alignments within the BSJs for each assembled circRNA (Fig. 1f). The resulting FSG is dissected into paths by using an adapted deep-first search method, which iteratively traverses from different source node to find all non-redundant paths (Additional file 1: Figure S6). Next, the following steps are to estimate the abundance of each circular isoform (Additional file 1: Figure S7). First, a Monte Carlo method is employed to simulate the distribution of BSJ reads on each path according to the insert length of RNA-seq library, which will be used to estimate the coverage of each node and edge of this path. Then, an approximate exhaustive search method is employed to find the optimum solution of the abundance of each path. Specifically, CIRI-full initially assigns a random value to each path and then calculates the abundance of every node and the splicing events of each edge on the path based on the simulated BSJ-reads distribution. Consequently, CIRI-full calculates the distance between the accumulated putative abundance of each node and splicing events of each edge. This distance score represents the discrepancy between the putative and real abundance of each path. To obtain the smallest distance, CIRI-full corrects the putative abundance of each path iteratively according to the FSG until the distance scores get converged. After iterative computation, the optimum solution will be output, and thus, the abundance of each path is determined (Fig. 1f).

### RO feature facilitates identification of low-abundance circRNAs

**Fig. 2:**
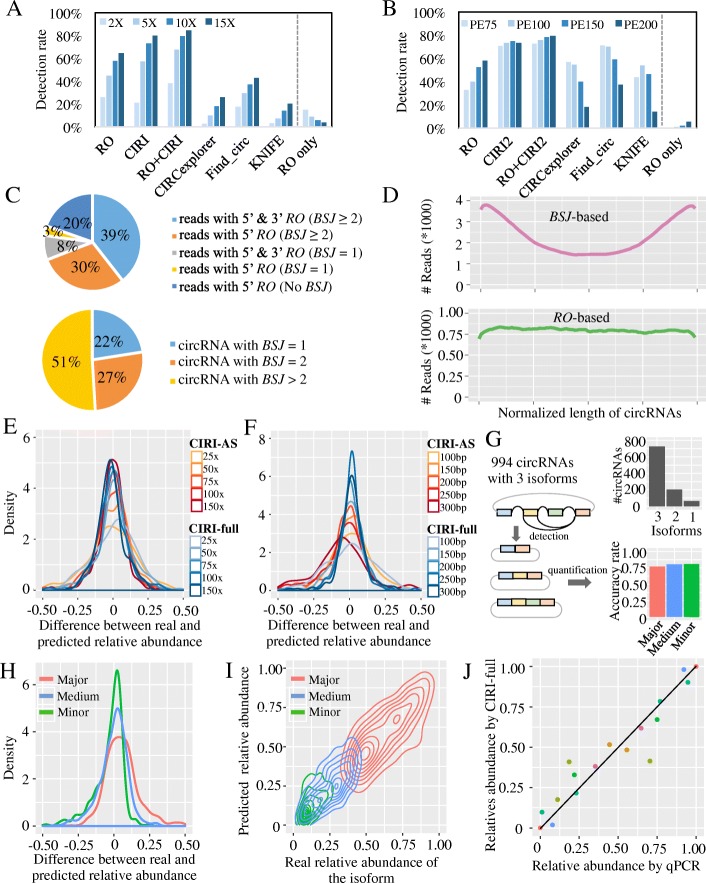
Performance evaluation of the RO approach to circRNA identification. **a**, **b** Performance comparison between the RO approach and the BSJ-based tools. “RO only” represents the circRNAs that are only identified by the RO approach. **a** CircRNA detection rate on the four data sets with different circRNA depth. **b** CircRNA detection rate on the four data sets with different read length. **c** Component of circular RNA and circular RNA reads detect by RO in simulated data (5X, paired-end 200 bp). **d** Base depth distribution of BSJ reads (pink) and RO reads (green) on normalized circular RNAs. **e**, **f** Accuracy evaluation of the AS events-based and the FSG-based quantification algorithms (CIRI-AS vs. CIRI-full) using simulated circRNA-containing transcriptomic data sets, including different sequencing depth (**e**) and different read length (**f**). **g** Sensitivity evaluation of the FSG-based quantification algorithm on simulated circRNA-containing transcriptomic data sets, where each circRNA contains three isoforms with different abundance. The bar plot on the right top displays the number of isoforms detected in 994 circRNAs; the bar plot on the right bottom shows the accuracy of FSG quantification in three types of isoforms. Accuracy rate is defined as the percentage of isoforms that are fully reconstructed and of which the predicted relative abundance matches the ground truth (difference between them is smaller than 20%). **h**, **i** The accuracy distribution of FSG method on the three types of reconstructed isoforms. **j** Experimental validation of the FSG-based isoform quantification algorithm. *X*- and *y*-axis represent the relative abundance of circRNA isoforms determined by qPCR and the FSG-based algorithm, respectively. Each dot represents a circRNA isoform, and dots in the same color represents that they come from the same circRNA

To explore the advantages of the RO feature, we extensively compared the performance of the RO-based method with that of BSJ-based methods by simulating circRNA-containing transcriptomic datasets. These datasets contained RNA-seq paired-end reads with an average library size of 350 bp and an average circRNA length of 300 ± 150 bp, where the length distribution was inferred from a HeLa circRNA dataset (Additional file 1: Figure S8). To measure the effect of read length on the results obtained using these two strategies, we simulated datasets with an average circRNA abundance of 10X and read lengths of 75, 100, 150, and 200 bp **(**Additional file 1: Figure S9). As expected, the sensitivity of the RO-based method increased significantly, considering that 75% of the simulated circular transcripts were shorter than 480 bp (Additional file 1: Figure S8A). Moreover, the number of circRNAs that were exclusively detected by the RO method also increased with increased read length. Surprisingly, this contrasted sharply with the performance of the BSJ-based tools except for CIRI2, for which the sensitivity dropped rapidly, especially with read lengths of 200 bp. The primary reason for this decrease is the increased number of junction sites produced by long reads. Most current BSJ-based approaches, except for CIRI2, are specifically designed for short reads, and they ignore the split alignment of long reads with more than three splitting sites. Next, we compared the performance of the RO- and BSJ-based methods in detecting low-abundance circRNAs. The simulator was adapted to generate paired-end reads with lengths of 200 bp and circRNAs with a gamma distribution of expression levels, in which most circRNAs exhibited low abundance (Additional file 1: Figure S9). As shown in Fig. 2a, b and Additional file 1: Figure S10, in all of the compared cases, the RO-based method achieved a sensitivity comparable to that of CIRI2 and a specificity similar to that of the BSJ-based methods. Notably, circRNAs that were only detected by the RO-based method were of low abundance, suggesting the potential application of the method for identifying low-abundance circRNAs.

To obtain a more comprehensive understanding of the RO feature, we further compared the relationship between RO reads and BSJ reads used for circRNA identification. As shown in Fig. 2c, 69% (30% + 39%) of the RO-merged reads were derived from 78% of the circRNAs (27% + 51%) having BSJ read support ≥ 2, and more than half of these RO-merged reads had 3′ RO also, thus indicating that they could directly generate full-length circRNA transcripts. The remaining RO-merged reads comprised the 22% of the circRNAs that were exclusively identified by the RO-based method, thus suggesting that the RO-based method offers a distinct advantage compared with previous BSJ-based methods, in which low-abundance circRNAs with limited BSJ read support are usually discarded by setting an arbitrary threshold (e.g., #BSJ reads > 2 or more). We further surveyed the base depth distribution of circRNAs and found that the RO reads produced a more uniform depth distribution along the normalized circRNA transcript than the BSJ reads (Fig. 2d). Based on the foregoing results, we conclude that the RO feature performs well in identifying low-abundance circRNAs and generating more uniform read distributions along circular transcripts. This improvement will greatly facilitate downstream reconstruction of full-length circRNAs.

### The FSG-based algorithm accurately identifies and quantifies circular isoforms

To validate the quantification accuracy of the FSG-based algorithm, we simulated circRNA-containing transcriptomic datasets with read length varying from 100 to 300 bp and sequencing depth at 25–150 fold (Fig. 2e, f**)**. We sought to evaluate the performance of this approach by comparing with CIRI-AS. Therefore, all the circRNAs in these data sets were designed to possess two isoforms, where CIRI-AS can estimate the relative abundance of the two isoforms within a certain circRNA using the PSI values. We then compared the accuracy of these two tools by calculating the discrepancy between predicted and real abundance of each isoform. As shown in Fig. 2e, both of these two approaches achieved a high level of accuracy, especially for those isoforms with an abundance over 50 fold. Moreover, the FSG-based quantification approach exhibited increased levels of accuracy with increasing read length, relative to the splicing events-based method (Fig. 2f). For more complicated splicing pattern, we further designed a simulated dataset containing 994 circRNAs with three isoforms, which were referred to as major, medium, and minor isoform according to their abundance, respectively. We performed CIRI-full on this dataset, detected circular isoforms within each circRNA, and determined their abundances. Among these simulated circRNAs, 73% (726/994) of them could be precisely recognized for all three isoforms. For isoform quantification, an average of 79% of these isoforms can be correctly determined (Fig. 2g–i). We further used four circRNA data sets with biological replicates and two simulated datasets to evaluate the reliability of isoform quantification by CIRI-full. As shown in Additional file 1: Figure S11, 76.2 ~ 85.6% of moderately or highly expressed isoforms (#BSJ reads > = 30) in the real datasets, including human brain tissue, human liver tissue, and Hs68 cell line, could be accurately quantified using CIRI-full. Similar findings were also found in the simulated datasets.

To experimentally evaluate our approach on quantifying circRNA isoforms, we performed real-time RT-PCR to validate eight randomly selected circRNAs with two or three isoforms from a transcriptomic data set of HeLa cells (Additional file 1: Figure S12–14). Each of these circRNAs was predicted to contain at least two isoforms. We designed 17 pairs of primers to amplify fragments containing both BSJ and alternatively spliced cirexons and quantified their abundance using real-time RT-PCR. As shown in Fig. 2j and Additional file 1: Table S1, the abundances of circRNAs determined by CIRI-full and qRT-PCR show a high level of consistency, demonstrating the reliability of the FSG-based method for isoform-level circRNA quantification.

### Reconstruction of full-length circRNAs based on CIRI-full

**Fig. 3:**
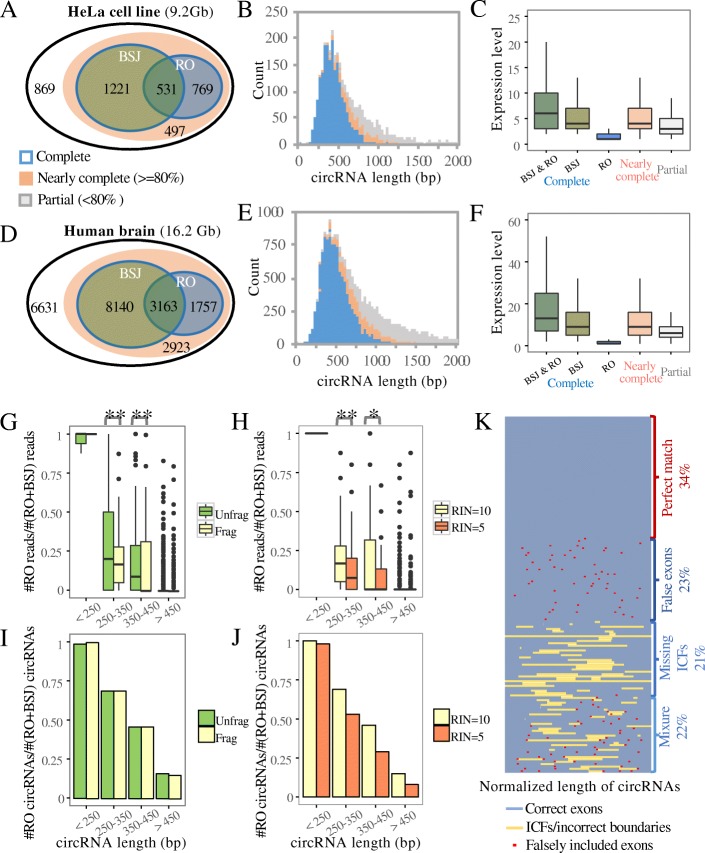
Full-length circRNA reconstruction of HeLa cell line (**a–c**) and human brain (**d–f**) transcriptomes with RNase R + RiboMinus treatment. **a**, **d** CircRNAs reconstructed using both the RO and BSJ features. Completely reconstructed circRNAs are shown in blue-lined ovals. Nearly complete and partial circRNAs are shown in orange and gray, respectively. **b**, **e** Length distribution of reconstructed circRNAs in the HeLa cell line and in human whole brain tissue. Complete, nearly complete and partial circRNAs are shown in blue, orange, and gray, respectively. The length of partially reconstructed circRNAs was estimated based on supported BSJ/RO reads and sequencing depth in the RNase R-treated sample. **c**, **f** Expression levels of different categories of circRNA. **g**–**j** Performance of the RO feature in circRNA identification when applied to fragmented or low-quality RNA samples. The green bar indicates the RNA data set derived from high-quality RNA (RIN = 10) without manual fragmentation. The yellow bar indicates the RNA data set derived from high-quality RNA (RIN = 10) with manual fragmentation. The red bar indicates the RNA data set derived from low-quality RNA (RIN = 5) with manual fragmentation. **g**, **h** Comparison of the ratio of RO reads to total circRNA reads for circRNAs of different lengths. ‘**’ and ‘*’ represent *P* < 0.01 and *P* < 0.05, respectively (Mann–Whitney *U* test). **i**, **j** Ratio of the number of circular RNAs with RO reads to total circular RNAs of a given length. **k** Comparison of full-length circRNA structure and corresponding annotated exon regions in human brain tissue. The 150 most highly expressed circRNAs that were completely reconstructed are shown. Each line represents a circRNA with normalized length

To further investigate the utility of using both RO and BSJ features in circRNA reconstruction, we generated 9.2 Gb of sequencing data with RiboMinus + RNase R treatment from HeLa cells. CIRI-full was then employed to identify circRNAs and to reconstruct their full-length transcripts. As shown in Fig. 3a, 77.6% of the circRNAs were identified as full- or nearly full-length circular transcripts, indicating a high efficiency of CIRI-full in reconstructing circRNAs by combining RO and BSJ features. We further explored the length distribution of these reconstructed circRNAs and found that the majority were between 150 and 500 bp in length (Fig. 3b). At this length interval, circRNAs can be well covered by long paired-end reads (e.g., PE250 or PE300), but above this length, it is difficult to recover their complete sequences. The lengths of the unconstructed circRNAs were estimated by summing their potential cirexons; the results indicated that their lengths ranged from 750 to 1250 bp (Additional file 1: Figure S14A). These circRNAs represented only one quarter of the total number of identified circRNAs. By examining the length distribution and the expression levels of full-length circRNAs in more detail, we found that the circular transcripts reconstructed by the RO and BSJ features exhibited distinct patterns. Specifically, the BSJ feature focused on long and highly transcribed circRNAs, whereas the RO feature preferentially identified short and low-abundance circRNAs, especially those with only one RO read support (Fig. 3c and Additional file 1: Figure S14A). This finding highlights the general principle that depending on the RO or BSJ feature, a single feature alone may not be sufficient to recover all full-length circRNAs.

For further validation, we generated 16.2 Gb of sequencing data from human brain samples based on RiboMinus RNA sequencing with RNase R treatment and performed CIRI-full on this dataset. Most of the circRNAs identified in this dataset were full- or nearly full-length (Fig. 3d). The number of circRNAs in the brain dataset was greater than that in HeLa cells when the sizes of the data sets were normalized. The length distribution of full-length circRNAs and the recognition patterns of the RO and BSJ features were similar to those identified in HeLa cells (Fig. 3e, f and Additional file 1: Figure S14B). Notably, a large majority of cirexons that were specifically identified by RO reads were enriched in both RiboMinus-treated and RiboMinus/RNase R-treated samples as compared to the poly (A) enrichment sample (Additional file 1: Figure S15).

To verify the reconstructed circRNAs based on RO features, both computational and experimental approaches were employed. Firstly, over 80% of circRNAs detected by the RO-based method could be supported by at least one BSJ-based method (Additional file 1: Figure S16A). The remaining circRNAs solely detected by the RO-based method also exhibited a typical reversely mapping signature when aligned them to the reference genome (Additional file 1: Figure S16B). Secondly, we performed experimental validation by randomly selecting 15 circRNA loci in the HeLa sample; 3, 6, and 6 of these loci were highly, moderately, and weakly transcribed, respectively. Outward-facing primers were designed to amplify fragments containing BSJs, and the sequences of the PCR products were determined via Sanger sequencing. As shown in Additional file 1: Figure S17–19, all 15 of the loci were successfully validated. For additional validation, six predicted full-length circRNAs were randomly selected and validated using the same approach. As shown in Additional file 1: Figure S20, all of these predicted full-length circRNAs were successfully verified using the experimental method. This solid evidence demonstrates the excellent reliability of CIRI-full in reconstructing full-length circRNAs.

To measure the robustness of using RO and BSJ features for circRNA reconstruction, RNA-seq libraries were constructed using both high-quality RNA (RNA Integrity Number, RIN = 10) and degraded RNA (RIN = 5) of HeLa cells treated by RNase R and RiboMinus. For the high-quality RNA sample, two different methods (with or without fragmentation) were used to construct RNA-seq libraries. For these three libraries, 9.2, 5.3, and 11.6 Gb of sequencing data were generated, respectively. We employed CIRI-full to detect RO and BSJ reads from these data; the ratio of RO reads to the sum of RO and BSJ reads was then calculated. As expected, compared with the unfragmented library, the RO reads ratio decreased considerably in the fragmented and low-quality libraries across all the compared distribution levels (Fig. 3g, h). In particular, the low-quality library, in which the circRNAs suffered from degradation, exhibited the lowest RO reads ratio. We next investigated whether the number of RO-identified circRNAs was also affected (Additional file 1: Figure S21). Therefore, we performed CIRI-full on each dataset and calculated the ratio of RO-identified circRNAs to the sum of RO- and BSJ-identified circRNAs. We found that although the ratio of RO reads decreased, the number of RO-identified circRNAs was unaffected by RNA fragmentation (Fig. 3i) and only weakly influenced by RNA degradation (Fig. 3j). These findings further confirm that the RO feature can be used to efficiently detect low-abundance circRNAs even if most of the RO reads are degraded or fragmented.

In addition to its high sensitivity and robustness in circRNA identification, CIRI-full also offers high accuracy for exploring detailed internal components within circRNAs. In contrast, previous studies simply combined all known or aligned mRNA exons in a sequential order as putative full-length circRNAs (hereafter referred to as the reference-based method). We measured the accuracy of full-length circRNAs predicted using the reference-based method by aligning them with the circular transcripts reconstructed using CIRI-full. As shown in Fig. 3k, only 34% of these predicted full-length circRNAs in human brain predicted by the reference-based method perfectly matched the circular transcripts reconstructed using CIRI-full, thus suggesting the former method’s low level of accuracy. The errors can be classified into three categories: false cirexons, missing ICFs, and a mixture of the former two errors (Additional file 1: Figure S22). False cirexons, representing the insertion of false additional exons, accounted for 23% of the predicted circRNAs, and the average number of false exons per circRNA was 2.4. Moreover, 21% of the errors were identified as missing ICFs, referring to missing intronic/intergenic circular fragments; these included an average of 25.5% of the full-length circular transcripts. A mixture of both false cirexons and missing ICFs accounted for up to 22% of the errors. A similar error rate was also consistently observed in HeLa cells and mouse brain samples (Additional file 1: Figure S23), strongly indicating that the reference-based method is error-prone and not reliable for resolving the internal structure of circRNAs. Compared with previous approaches, which focused on the determination of internal sequence or alternative splicing events, CIRI-full exhibited a high efficiency on reconstructing circular transcripts using the combination of RO and BSJ features (Additional file 1: Figure S24).

### Profiling full-length circular RNAs in vertebrate brains

**Fig. 4:**
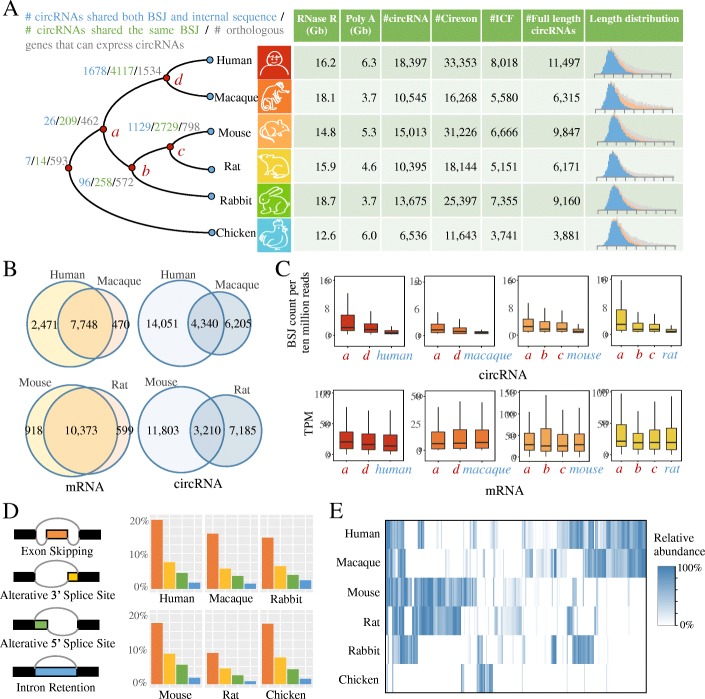
CircRNAs expression profiles in vertebrate brain tissues. **a** CircRNAs identified in six vertebrate brain tissues (RNase R + Ribomiuns treatment) by CIRI-full. The number of shared circRNAs is shown on the phylogenetic tree. The table on the right shows the RNA-seq data set size and the numbers of identified circRNAs, cirexons, intronic/intergenic circRNA fragments (ICFs), and full-length circRNAs. The histogram on the right shows the length distribution of reconstructed circRNAs; blue, orange, and gray represent complete, nearly complete and partial circRNAs, respectively. **b** Overlap of highly expressed mRNAs and circRNAs in closely related species. Obviously, mRNA expression is more conserved than circRNA expression in closely related species (human vs. macaque, mouse vs. rat). **c** Expression levels of circRNAs and their corresponding mRNA genes. *a*, *b*, *c*, and *d* represent the four ancestral nodes, as shown in panel **a**. Species-specific circRNAs in four species (shown in blue) have much lower expression levels than the shared circRNAs present in ancestral nodes. **d** Percentage of circRNAs (BSJ ≥ 10 reads) containing four types of alternative splicing events. **e** Expression profiles of circRNA isoforms in the six species. The relative abundance of circRNA isoforms were normalized between 0 and 1

Evolutionary analysis is essential for insights into the genetic basis of phenotypes and into functional screening. For circRNAs, such analyses remain scarce despite growing attention to these circular transcripts. With this goal in mind, we determined circRNA repertoires in brain samples of six vertebrate species, including human, macaque, mouse, rat, rabbit, and chicken. For each species, a whole-brain sample was sequenced using RNA-seq with RiboMinus/RNase R treatment, Poly A enrichment and RiboMinus treatment (Fig. 4a). Then, we applied CIRI-full to the RiboMinus/RNase R-treated transcriptomic data to comprehensively explore the circular transcripts for each species, including the numbers of circRNAs, cirexons, BSJ reads, ICFs, full-length circRNAs, and RO reads. HISAT and StringTie [41] were performed on the Poly (A) enrichment data to obtain linear transcript abundance. We identified approximately 3500 to 11,500 full-length circRNAs in these organisms. Analysis of the lengths of these full-length circular transcripts revealed that circRNA length was highly conserved among these species, with the majority of circRNAs ranging from 250 to 500 bp in length (Fig. 4a). Next, we measured the exon boundary conservation of orthologous circular and linear transcripts between pairs of closely related species, including human and macaque and mouse and rat. Considering that most of the cirexons in circRNAs are identical to those in linear mRNAs, only the ICFs that are exclusively present in circRNAs were used for boundary conservation analysis. As shown in Additional file 1: Figure S25, lncRNA exon boundaries exhibited larger and more frequent changes across mammals than did protein-coding exons. Interestingly, the boundary conservation level of ICFs was similar to that of protein-coding exons. Consequently, compared with lncRNAs, circRNAs exhibit more constraint with respect to maintaining an exact position of splicing events among orthologous pairs. We further counted the number of shared circRNAs and mRNAs in these two pairs of species and found that circRNAs exhibited significantly decreased conservation compared with protein-coding genes (Fig. 4b); only a small subset of orthologous circRNAs were conserved between closely related species. For example, approximately 23.5% of human circRNAs were also expressed in macaque and 21.4% of mouse circRNAs were also expressed in rat, whereas more than 76% of protein-coding genes were expressed in both members of these two species pairs.

We further examined the orthologous circRNAs present in these six vertebrates and calculated the number of orthologous circRNAs that possessed the same sequences and BSJs and the number of shared genes on their ancestral nodes (denoted by “*a*”, “*b*”, “*c*”, and “*d*”). As shown in Fig. 4a, the number of shared full-length orthologous circRNAs and BSJs decreased rapidly with increased genetic distance. In contrast, the number of shared orthologous circRNAs decreased much more slowly, thus indicating that although derived from the same genes, the circRNAs of different species diverged rapidly in terms of sequences and BSJs. Next, the expression levels of orthologous circRNAs and genes on each node were estimated. As shown in Fig. 4c, the shared orthologous circRNAs exhibited increased levels of expression compared with lineage-specific circRNAs, whereas this scenario was not observed in the shared orthologous genes from which the circRNAs were derived. These findings suggest that there is a distinct evolutionary conservation pattern of orthologous circRNAs and that these shared circRNAs provide valuable targets for further functional screening. We next surveyed circRNA AS events in the six species. All four types of AS events could be detected within circRNAs in all these species (Fig. 4d). Exon skipping (ES) was the most prevalent AS type in circRNAs. Alternative 3′-splicing site (A3SS) and alternative 5′-splicing site (A5SS) were also major circular AS types, in agreement with our previous study showing that AS events not only occur in mRNAs but are also prevalent in circRNAs [10]. To this end, we investigated the expression level of conserved circRNA isoforms in these species. As shown in Fig. 4e, conserved circRNA isoforms exhibited similar splicing patterns in closely related species. For example, the expression and splicing patterns of these circRNAs were more similar between human and macaque than between human and other species.

### Read length is a key determinant of circRNA identification but not quantification

**Fig. 5:**
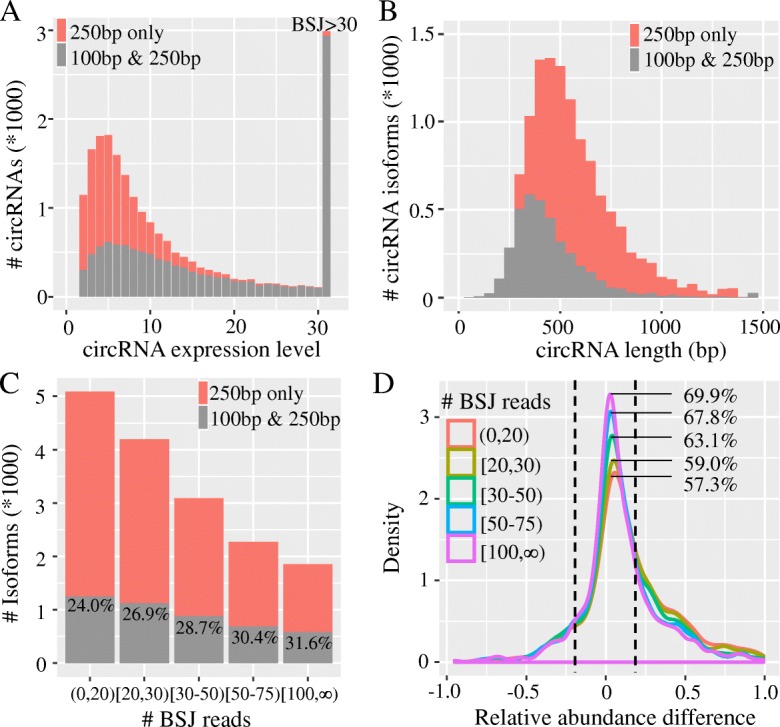
Sequencing length affects circRNA identification but not quantification. Gray bars represent the circRNAs that could be identified from both datasets and red bars represent the circRNAs exclusively detected in the PE250 dataset. **a** The number of circRNAs detected by CIRI_full from the PE250 and PE100 datasets. **b** The length distribution of reconstructed circRNA isoforms. **c** The number of reconstructed isoforms with different expression levels. **d** The difference of relative expression levels of circRNA isoforms estimated from PE250 and PE100 datasets. Different circRNA expression levels are shown in different colors. Two vertical dashed lines represent the threshold of relative abundance difference between PE100 and PE250 (± 0.2). The ratios in the panel represent the percentage of accurately quantified circRNA isoforms

Considering that most of publicly available RNA-seq data sets are generated for linear transcripts, which are typically in short sequencing length and contain a very limited fraction of RO reads, one may question whether this FSG quantification method works on short sequencing reads. To test this possibility, we truncated the 250-bp paired-end reads from human brain RNA-seq data set (RNase R + RiboMinus treatment) to 100-bp paired-end reads, and compared the performance of CIRI-full on both data sets with long (PE250) and short (PE100) sequencing reads. As shown in Fig. 5a, although most of the highly expressed circRNAs (BSJ reads > = 20) could be successfully identified in both data sets, the number of identified circRNAs transcribed at low levels decreased rapidly after truncating into short reads. This scenario was also observed for the assembled circular isoforms in terms of number and length (Fig. 5b). In particular, only less than a half of full-length circRNA isoforms with length > 300 bp were reconstructed by the FSG-based method. It has been demonstrated that the number of assembled full-length linear transcripts can benefit from increasing sequencing depth [43]. We therefore investigated whether this holds true for circRNA isoforms. We classified the identified circRNAs into five categories according to their expression level and subsequently calculated the number of full-length circRNA isoforms in each category. Strikingly, the recovery of full-length circRNA isoforms did not considerably improved with increasing sequencing depth (Fig. 5c), indicating that the read length rather than sequencing depth is the key determinant of circRNA isoform detection. This finding further raised the question as to whether the read length affects the accuracy of circRNA isoform quantification. We checked the relative abundance difference of the shared circRNA isoforms in these two data sets (PE250 and PE100) and found a high level of concordance, especially for highly expressed circRNAs (Fig. 5d). This observation demonstrates that the FSG-based method is efficient in quantifying circRNA isoforms even with short read length, but researchers can recognize more circRNA isoforms by increasing the sequencing read length.

### Isoform-level quantification helps filter false positives in differential circRNA expression analysis

**Fig. 6:**
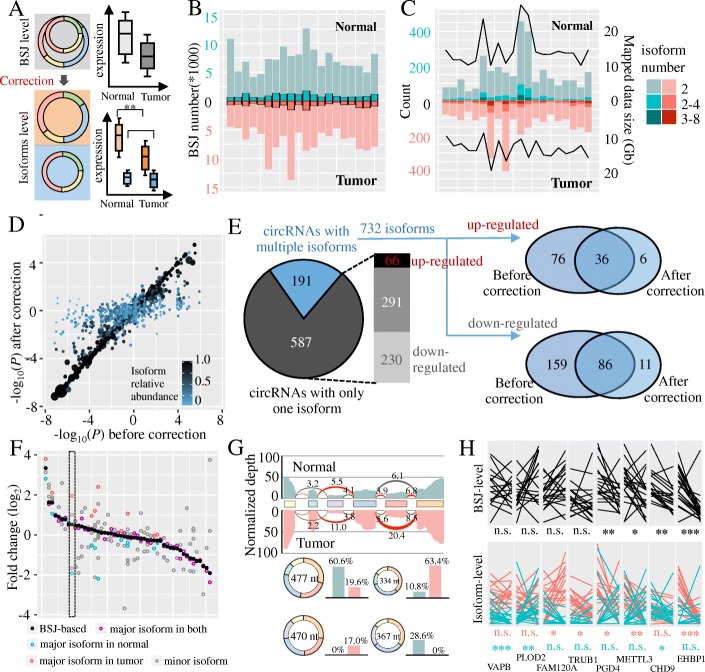
Differential circRNA isoform expression between normal and tumor liver tissues of 20 HCC patients. **a** A schematic comparison between BSJ-level and isoform-level differential expression analysis. **b** The number of circRNAs detected from normal and tumor liver tissues of 20 HCC patients. Bars in light color represent the numbers of circRNAs in a certain sample, and bars in dark color represent the numbers of highly expressed circRNAs (> 1 BSJ read per 10 million reads). **c** The number of circRNAs containing AS events that are detected by CIRI-AS. Light bars represent the circRNAs with one AS event and dark bars correspond to circRNAs containing more than one AS events. Black curved lines represent the total mapped data size for each sample. **d** Comparison of differential expression analysis between BSJ level (*x*-axis) and isoform level (*y*-axis). Each dot denotes a circRNA isoform, with its size representing the expression level and its color representing its relative abundance in the parental circRNA. **e** circRNAs in panel **d** can be classified into circRNAs with only one isoform and circRNAs with multiple isoforms. For circRNAs with multiple isoforms, Venn diagrams show the discrepancies of significantly up- or downregulated isoforms between BSJ level and isoform level differential expression analyses. **f** The average isoform expression fold change between normal and tumor tissues of the top 50 most highly expressed circRNAs that have multiple isoforms. Black dot represents the average fold change of circRNAs at the BSJ-level quantification. The dashed box highlights an example shown in panel **g**. **g** An example of alternative splicing switch between normal and tumor samples. CircRNA (chr2:207144264|207162097) locating on the ZDBF2 gene can express four isoforms. Rectangles with different colors represent the cirexons within this circRNA. The green and red histograms on each cirexon represent the normalized sequencing depth in normal and tumor samples, respectively. The curve connected cirexons represents the forward splice junction (FSJ) within this circRNA, and its width is proportional to the read support. The red curve represents the FSJ of the dominant circular isoform. The relative abundance of four circular isoforms is shown in bar plot (bottom). **h** Expression profiles of eight circRNAs quantified at BSJ and isoform level between normal (left) and tumor (right) tissues across 20 HCC patients. Red and cyan lines represent the expression profile of the major and minor circRNA isoform, respectively. All statistic significances are calculated by Mann–Whitney *U* test. “**” and “*” represent *P* < 0.01 and *P* < 0.05. “n.s.” indicates “not significant”

Unlike mRNA transcripts, current differential expression analysis on circRNAs is limited to the BSJ level due to the inability of detecting isoforms within a certain BSJ (Fig. 6a, top). Using our FSG-based isoform quantification algorithm, however, it is possible to distinguish differentially expressed isoform from circRNAs with the same BSJ (Fig. 6a, bottom). To explore the difference of expression patterns between BSJ and isoform levels, we applied CIRI-full to 40 RNA-seq data sets of HCC tumor tissues and their adjacent normal tissues [42]. The sequencing data size mapped to the reference genome varied from 10 to 27 Gb and the number of identified circRNAs from these data sets ranged from approximate 4000 to 14,000 (Fig. 6b). Notably, a small fraction of circRNAs in these 40 samples contained at least two isoforms. Moreover, the number of isoforms positively correlated with sequencing data size, suggesting that increasing sequencing depth and read length should facilitate the detection of isoforms within circRNAs (Fig. 6c).

To better understand the discrepancy between BSJ-level and isoform-level differential expression analysis, we extracted top 1000 most abundant circRNAs that expressed in at least 80% of the 40 samples and found that 778 of them could be fully reconstructed. Then, we investigated their expression changes between normal and tumor tissues at both BSJ and isoform levels. Specifically, Mann–Whitney *U* test was employed to calculate the significance of expression alternation and signs of *P* value were used as proxies for directions of changes in expression (Fig. 6d). Among these 778 circRNAs, 587 of them showed the same significance values between BSJ-level and isoform-level differential expression analysis, because each of these circRNAs only expressed a single isoform, with 66 and 230 significantly up- and downregulated in tumor tissues, respectively **(**Fig. 6e**)**. Regarding the remaining 191 circRNAs that expressed multiple isoforms, the significance level of their differential expression was generally overestimated because different isoforms of circRNAs with a certain BSJ cannot be distinguished from each other if they are quantified at the BSJ level. After corrected for isoform-level quantification, only 32% of them were still significantly upregulated in tumor samples. The same phenomenon was also observed in downregulated circRNAs, where only 35% of them were kept after correction. Notably, a small number of circRNAs were recognized as differentially expressed isoforms only by the isoform-level quantification, indicating that other isoforms within the same BSJ may interfere with the performance of differential expression analysis solely based on the BSJ-level quantification.

To further investigate whether there were alternative splicing isoform switches present between normal and tumor samples, we extracted the top 50 most highly expressed circRNAs with multiple isoforms and calculated the expression fold change for each isoform between tumor and normal samples. We found that 10 out of 50 circRNAs underwent isoform switches, where the striking expression changes occur in the most abundant isoform (green and red circles, Fig. 6f). In contrast, the BSJ-level differential expression analysis cannot distinguish such scenario. For example, circRNA chr2:207144264|207162097 locating on the ZDBF2 gene could express four circular isoforms (Fig. 6g). The alignments of BSJ reads on the second and fourth cirexons, as well as the splicing events between the fourth and sixth exons, exhibited distinct read supports between tumor and normal samples, indicating the existence of alternative splicing isoform switches. Indeed, the circular isoform with sequence length of 477 nt was the dominant isoform in normal samples, whereas its expression dropped rapidly in tumor samples and the expression of this circRNA was dominated by the circular isoform with sequence length of 334 nt. Moreover, eight circRNAs were illustrated to serve as examples to detail the expression changes at both the BSJ and isoform levels between normal and tumor tissues of 20 patients (Fig. 6h). Although four of these circRNAs were found to be differentially expressed by the traditional BSJ-level quantification, it could not distinguish the real differentially expressed isoforms. In addition, there were also a few cases that certain circular isoforms exhibited increased expression levels across tumor samples, which were missed by the BSJ-level differential expression analysis. Collectively, these results highlight the limitation of BSJ-based quantification and the necessity of extending differential circRNA expression to the isoform level.

## Discussion

**Fig. 7:**
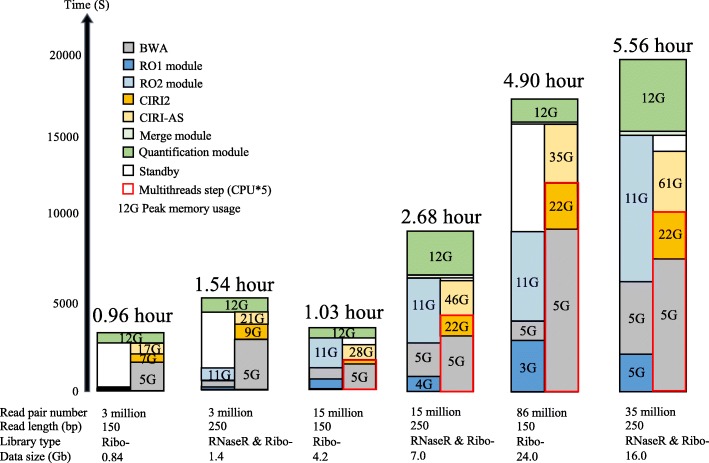
Time and memory usage of CIRI-full on human brain RNA-seq data sets. The CIRI-full pipeline consists two components, one is BSJ detection using CIRI2/CIRI-AS, the other is RO detection, and both are executed simultaneously. Height of boxes represents the running time of each module in this pipeline. Options “-t 5” was used for the last four datasets to activate the multithreading function of CIRI2 and BWA

This study presents a novel experimental and bioinformatic framework, CIRI-full, that can be used to efficiently reconstruct full-length circRNAs and quantify their expression at the isoform level from transcriptomic data. The main advantage of CIRI-full is that it utilizes an unfragmented library preparation approach to generate RO reads for circRNAs. To fully utilize these RO reads, we developed a new computational algorithm with the aim of reconstructing the complete sequences of circRNAs and circular isoforms within them and further proposed a new Forward Splice Graph (FSG)-based algorithm for isoform-level circRNA quantification. Through extensive evaluations of both simulated and real data sets from HeLa cells and from the human brain, we demonstrated that CIRI-full offers excellent performance in full-length circRNA reconstruction and isoform-level quantification. By applying this tool to brain samples of six vertebrate species, we demonstrated the reliability of CIRI-full on exploring comprehensive circRNA repertoires across multiple species and unveiling their evolutionary conservation and divergence. Further application of CIRI-full on human normal and tumor tissues, we systematically compared the difference between the BSJ-level and isoform-level differential expression analyses. We have packed CIRI-full with our previous tools (CIRI2 and CIRI-AS), and users can identify circRNAs, detect alternative splicing events, and reconstruct circular isoforms from transcriptomic data using a single command. The running time and peak memory usage of CIRI-full under different conditions are shown in Fig. 7. This tool will greatly accelerate our understanding the diversity and function of circRNAs, which will undoubtedly contribute to the field of circRNA studies.

Most approaches to circRNA identification rely exclusively on recognizing BSJs, and these methods are of limited application in determining the internal structure of circRNAs. In this study, for the first time, we propose an RO feature-based method for circRNA detection. This new approach is an important addition to the BSJ feature-based method, with each approach having its own advantages and limitations. Compared with the BSJ feature-based method, the RO feature-based method has the following distinct advantages. First, the RO feature provides more solid evidence for identifying full-length circRNAs. This greatly facilitates genome-wide full-length circRNA identification and thereby offers an indispensable advantage for downstream analyses, including functional and evolutionary analyses. Second, the RO feature facilitates the detection of weakly transcribed circRNAs, and even extremely low-abundance circRNAs could be efficiently and accurately identified. For instance, in the HeLa cell dataset, 3887 circRNAs were identified; 14.4% of these were supported by only one read, and 92% were successfully identified by CIRI-full. Considering that a vast majority of circRNAs in various transcriptomes are in low expression levels, the detection of such low-abundance transcripts is extremely important in exploring circRNA profiles. For example, when detecting circRNAs from RNA-seq data sets without RNase R treatment, most of the circRNAs are in low abundance compared with their linear counterparts. Therefore, the ability of identifying and reconstructing low-abundance circRNAs is not trivial in circRNA studies. Third, compared with BSJ reads, RO reads produce a more uniform depth distribution along the normalized circRNA transcript, which greatly facilitates quantification of circRNAs and determination of their internal structures. Compared with the BSJ feature, RO-based circRNA identification also has limitations. First, the RO-based approach requires longer reads to obtain an entire circRNA sequence. Considering that most circRNAs are between 200 and 800 bp in length, it should be possible to easily obtain RO reads as sequencing technology continues to advance. Second, a large library size is required to produce high-quality RO reads during library preparation.

Considering the potential significance of the biogenesis and functions of full-length circRNAs, several computational algorithms have been developed to determine the internal components of these circular transcripts. The reference-based prediction method has been revealed to be error-prone; up to 66% of the predicted full-length circRNAs in our study were demonstrated to contain errors, including false cirexons and missing ICFs. Despite the fact that spliced junction signature-based methods such as CIRI-AS represent an important step forward by facilitating accurate cirexon identification, full-length prediction of circular isoforms with complicated AS events is still not feasible. An alternative approach involves utilization of new sequencing technologies such as PacBio long read sequencing, which promises increases in read length of several orders of magnitude, thereby making full-length circRNA identification considerably easier. However, this is achieved at the expense of higher cost per base and lower throughput. Furthermore, it is not cost-effective to use PacBio long reads to obtain the complete sequences of circRNAs, especially considering that their lengths range from 250 to 800 bp. In contrast, CIRI-full generates high-throughput “long” reads that are sufficient for determining the complete sequences of most full-length circRNAs in an economical and practical manner. Without the need for additional library preparation and with bypassing of the fragmentation step, RO reads are easily obtained using general paired-end sequencing, greatly expanding the applicability of this method.

Besides circular transcript reconstruction, CIRI-full is also the first tool to provide isoform-level quantification for circRNAs. Previous studies revealed the prevalence of AS events within circRNAs and the importance of accurate quantification of circRNA expression to confirm their crucial functions in many biological processes. However, current state-of-the-art transcript quantification approaches largely focus on linear RNAs, which estimate expression abundance at both gene and transcript levels. Till now, there is no available method for isoform-level circRNA quantification. In this study, CIRI-full can successfully determine the abundance of circRNAs at both BSJ and isoform levels by employing the FSG-based algorithm, which reconstructs all full-length isoforms within each assembled full-length circRNA. By applying this approach to 40 RNA-seq data sets of human HCC tumor tissues and their adjacent normal tissues, we identified thousands of circRNAs of which most were assembled into full-length, and we systematically explored the discrepancy between BSJ-level and isoform-level differential expression analysis. We found that the significance level for differential expression of most circRNAs that expressed multiple isoforms was generally overestimated at the BSJ level. A large majority of differentially expressed circRNAs measured at the BSJ level tended to be false positives, as BSJ-level quantification cannot make a distinction among different isoforms within a certain circRNA. Consequently, for circRNAs with multiple isoforms, BSJ-level quantification does not necessarily reflect the real expression changes of certain circular isoforms between normal and tumor samples. These findings not only provide more accurate candidates for functional screening, but also unveil the complexity of circRNA isoform expression.

## Conclusion

This study, for the first time, presents a high-throughput approach, CIRI-full, that employs a new feature for full-length circRNA reconstruction and isoform-level quantification. Extensive evaluations demonstrate that CIRI-full exhibits excellent performance in circRNA identification and whole-sequence assembly, as well as isoform reconstruction and quantification. We applied CIRI-full to investigate the evolutionary conservation of circRNAs in brain transcriptomes across six vertebrates. In addition, we systematically compared the difference between the BSJ-level and isoform-level differential expression analyses using human liver tumor and normal tissues and found that a large majority of differentially expressed circRNAs measured at the BSJ level tended to be false positives. For circRNAs with multiple isoforms, isoform-level quantification instead of BSJ-level quantification can reflect the real expression changes of certain circular isoforms between normal and tumor samples. This study provides an indispensable approach for circRNA transcript reconstruction and quantification and highlights the necessity of deepening circRNA studies to the isoform-level resolution.

## Availability and requirements

The availability and requirements are listed as follows:

Project name: CIRI-full.

Project home page: https://sourceforge.net/projects/ciri.

Operating system(s): Linux, Mac.

Programming language: Java, Perl.

## Additional file

Additional file 1: **Figure S1.** Workflows of the RO detection method. **Figure S2.** All possible scenarios on the presence of RO and BSJ in RNA-seq reads. **Figure S3.** 5′ RO candidate read mapping and filtering steps. **Figure S4.** Full-length circRNA reconstruction. **Figure S5.** CircRNAs that cannot be reconstructed into full length. **Figure S6.** Workflow of the adapted DFS method in the FSG algorithm. **Figure S7.** Workflow of approximate exhaustive search in the FSG algorithm. **Figure S8.** Characteristics of simulated data sets. **Figure S9.** Abundance distributions of circRNAs in simulated data sets. **Figure S10.** False discovery rate of different circRNA detection tools. **Figure S11.** CircRNA quantification in four real RNA-seq data sets with biological replicates and two simulated datasets. **Figure S12.** Verified circRNA structure and their predicted relative abundance in total RNA-seq data set (PE100). **Figure S13.** Verified circRNA structure and their predicted relative abundance in total RNA-seq data set (PE250). **Figure S14.** Length of circRNAs in human HeLa cell line. **Figure S15.** Cirexon enrichment rate. **Figure S16.** RO feature is reliable in detecting lowly expressed circRNAs. **Figure S17.** Experimental validations of the RO method on detecting highly expressed circRNAs (# BSJ reads > = 30) in HeLa cell line. **Figure S18.** Experimental validations on moderately- expressed circRNAs (# BSJ reads > = 10 & < 30). **Figure S19.** Experimental validations on weakly-expressed circRNAs (# BSJ reads < 10). **Figure S20.** Experimental validations on reconstructing full-length circRNAs in HeLa cell line. **Figure S21.** RNA degradation and fragmentation can reduce the abundance of RO reads. **Figure S22.** Four examples of circRNAs in Fig. 3k. **Figure S23.** Comparison of full-length circRNA structure and corresponding annotated exon regions in human HeLa cell line (A) and mouse brain tissue (B). **Figure S24.** Performance comparison between CIRI-Full and CIRCexplorer2. **Figure S25.** Boundary conservation of orthologous exons in mRNA, circRNA and lincRNA. **Table S1.** RT-PCR and CIRI-full quantification results. (PDF 4930 kb)
