# Supplementary material for: Reconstruction of full-length circular RNAs enables isoform-level quantification
Source: Genome Med. 2019 Jan 19;11:2. doi: 10.1186/s13073-019-0614-1 (PMC6339429; doi:10.1186/s13073-019-0614-1)
Supplement: Supplementary file 1 — Figure S1. Workflows of the RO detection method. Figure S2. All possible scenarios on the presence of RO and BSJ in RNA-seq reads. Figure S3. 5′ RO candidate read mapping and filtering steps. Figure S4. Full-length circRNA reconstruction. Figure S5. CircRNAs that cannot be reconstructed into full length. Figure S6. Workflow of the adapted DFS method in the FSG algorithm. Figure S7. Workflow of approximate exhaustive search in the FSG algorithm. Figure S8. Characteristics of simulated data sets. Figure S9. Abundance distributions of circRNAs in simulated data sets. Figure S10. False discovery rate of different circRNA detection tools. Figure S11. CircRNA quantification in four real RNA-seq data sets with biological replicates and two simulated datasets. Figure S12. Verified circRNA structure and their predicted relative abundance in total RNA-seq data set (PE100). Figure S13. Verified circRNA structure and their predicted relative abundance in total RNA-seq data set (PE250). Figure S14. Length of circRNAs in human HeLa cell line. Figure S15. Cirexon enrichment rate. Figure S16. RO feature is reliable in detecting lowly expressed circRNAs. Figure S17. Experimental validations of the RO method on detecting highly expressed circRNAs (# BSJ reads > = 30) in HeLa cell line. Figure S18. Experimental validations on moderately- expressed circRNAs (# BSJ reads > = 10 & < 30). Figure S19. Experimental validations on weakly-expressed circRNAs (# BSJ reads < 10). Figure S20. Experimental validations on reconstructing full-length circRNAs in HeLa cell line. Figure S21. RNA degradation and fragmentation can reduce the abundance of RO reads. Figure S22. Four examples of circRNAs in Fig. 3k. Figure S23. Comparison of full-length circRNA structure and corresponding annotated exon regions in human HeLa cell line (A) and mouse brain tissue (B). Figure S24. Performance comparison between CIRI-Full and CIRCexplorer2. Figure S25. Boundary conservation of orthologous exons in mRNA, circRNA [file 13073_2019_614_MOESM1_ESM.pdf]

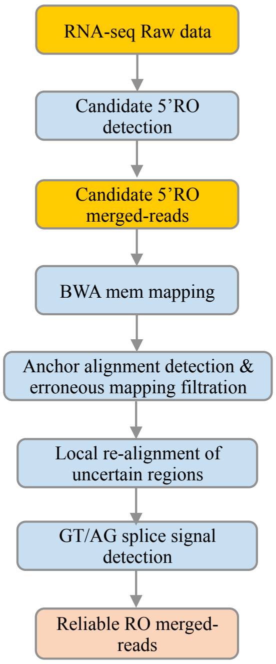

Figure S1. Workflow of the RO detection method.

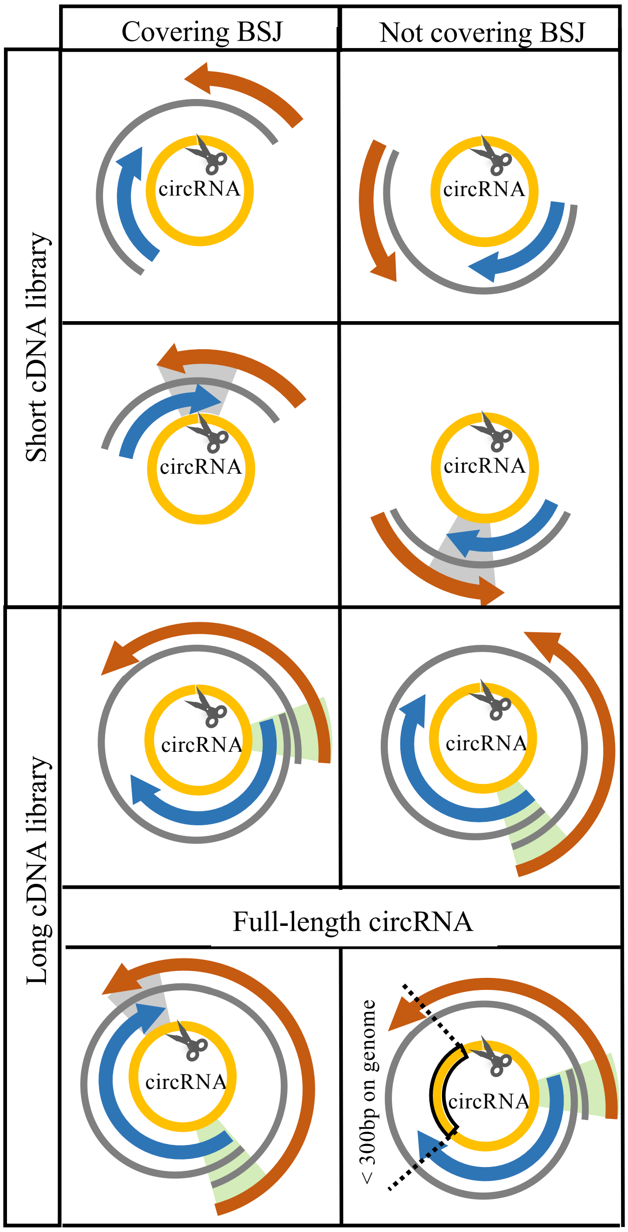

FigureS2. All possible scenarios on the presence of RO and BSJ in RNA-seq reads. The position of the scissor represents BSJ. Gray arc represents a cDNA fragment. Blue and red arrows represent paired-end reads. Gray zone represents 3'RO region and green zone represents 5'RO region. Black box in the bottom right figure represents a recovered cirexon from the alignment.

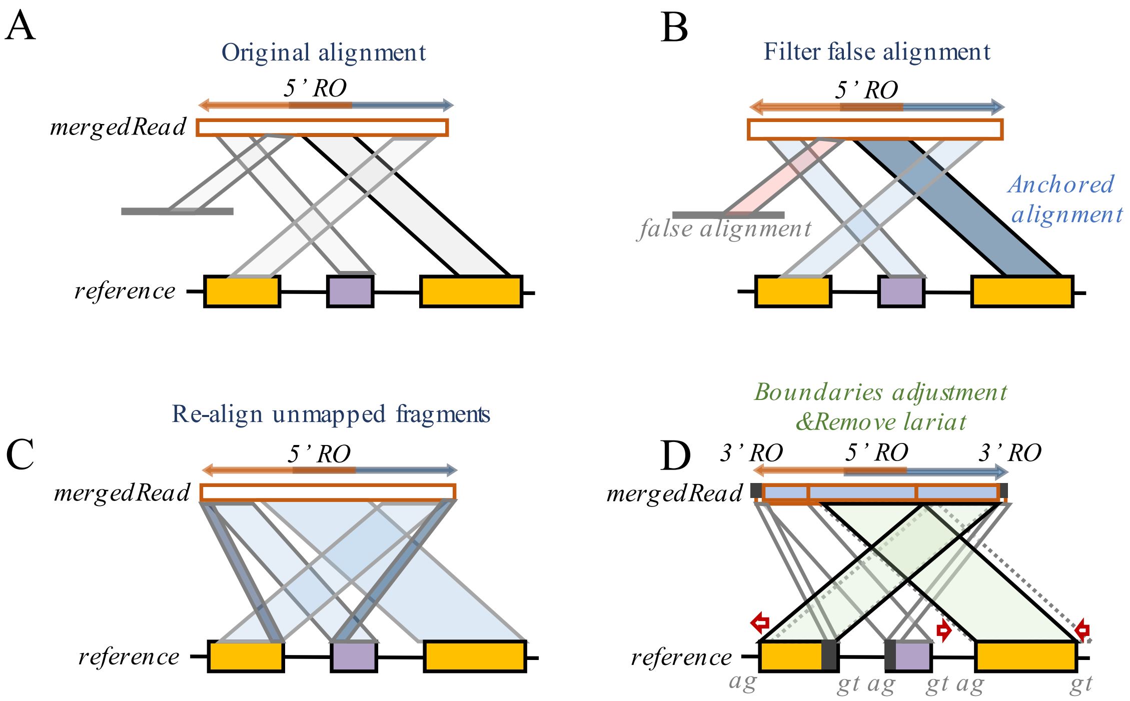

FigureS3. 5' RO candidate read mapping & filtering steps. (A) Raw alignment of RO merged reads on the reference genome. (B) The longest alignment is selected as an anchor. Alignments that are far away from this anchor are removed. (C) Re-align the unmapped fragments of the merged-read to the region around the anchor. (D) Refine the boundaries of all alignments based on GT/AG splicing signal. Merged-reads will be removed if one of the boundaries do not have splicing sites or cannot be fully aligned to the reference.

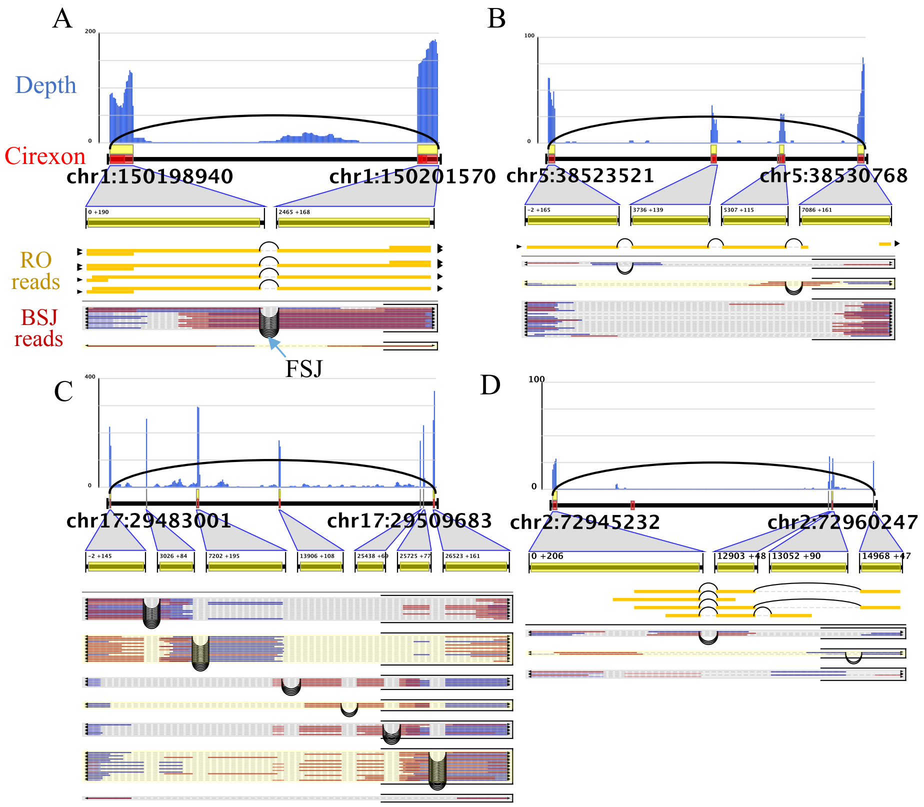

Figure S4. Full-length circRNA reconstruction based on RO and/or BSJ features. RO reads are shown as orange lines and BSJ read pairs are shown as blue/red lines. Black curved lines indicate FSJ. (A) RO reads with 5' & 3' RO can cover the full length of circRNA. (B) RO reads without 3'RO can be reconstructed into full-length circRNA when the mapping positions of its two terminals locate within a known cirexon. (C) A full-length circRNA will be reconstructed when all the FSJs within a BSJ can be continuously connected. (D) Both BSJ read pairs and RO reads are combined to complement each other and to reconstruct a full-length circRNA.

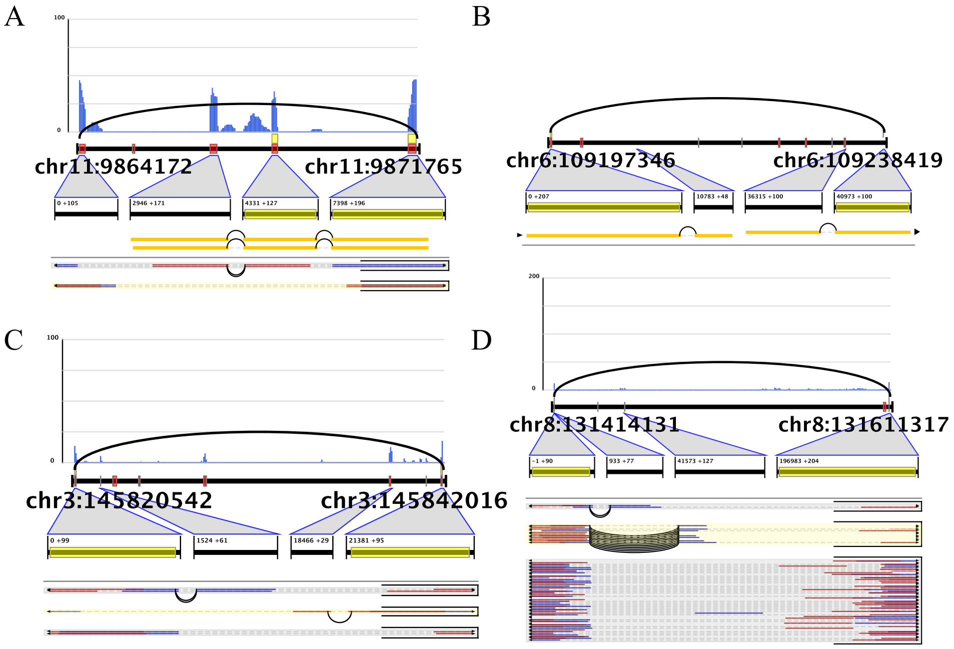

Figure S5. CircRNAs that cannot be reconstructed into full length based on RO and BSJ features. RO reads are shown as orange lines and BSJ read pairs are shown as blue/red lines. Black curved lines indicate FSJ. (A, B) RO reads cannot cover all FSJ events and only partial sequence of circRNA can be reconstructed. (C, D) For certain circRNAs without RO reads support, BSJ read pairs alone cannot cover full length circRNA for two following reasons: low sequencing depth (C) and long circRNA sequence (D).

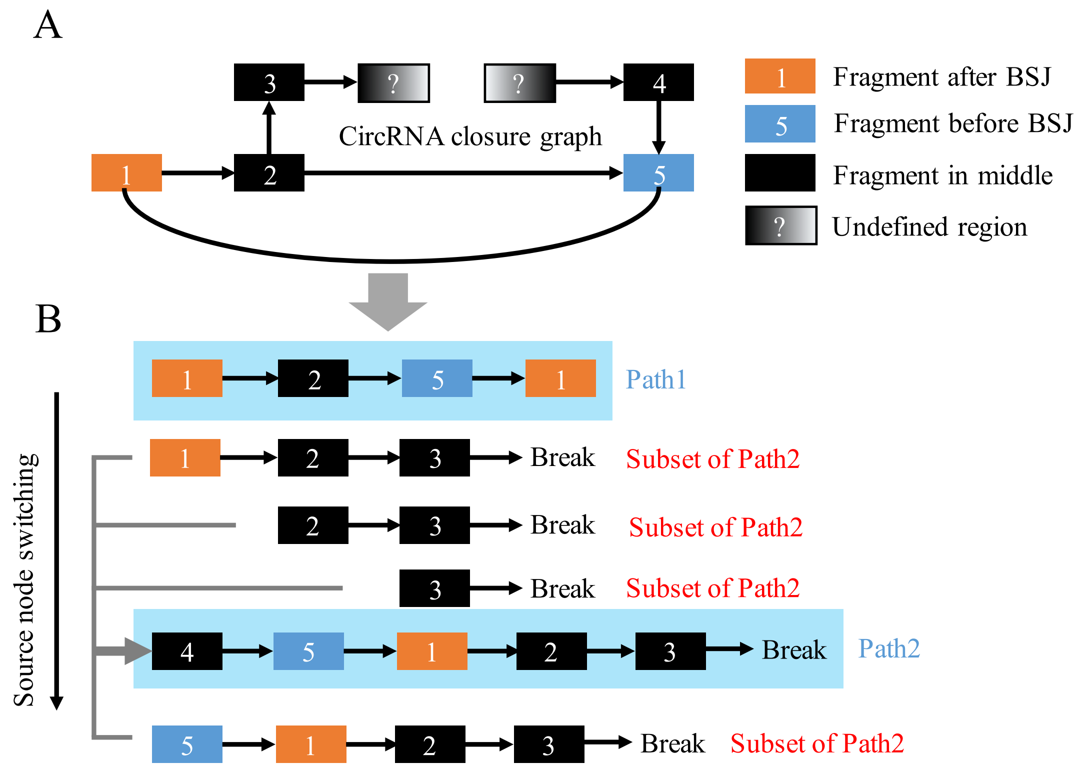

Figure S6. Workflow of the adapted DFS method in the FSG algorithm. (A) An example of circRNA Forward Splice Graph (FSG). Note that low coverage will cause a breakpoint where no splicing event can support the boundary of fragments between cirexon 3 and cirexon 4. (B) Adapted DFS algorithm switches the source node to find all the nonredundant paths.

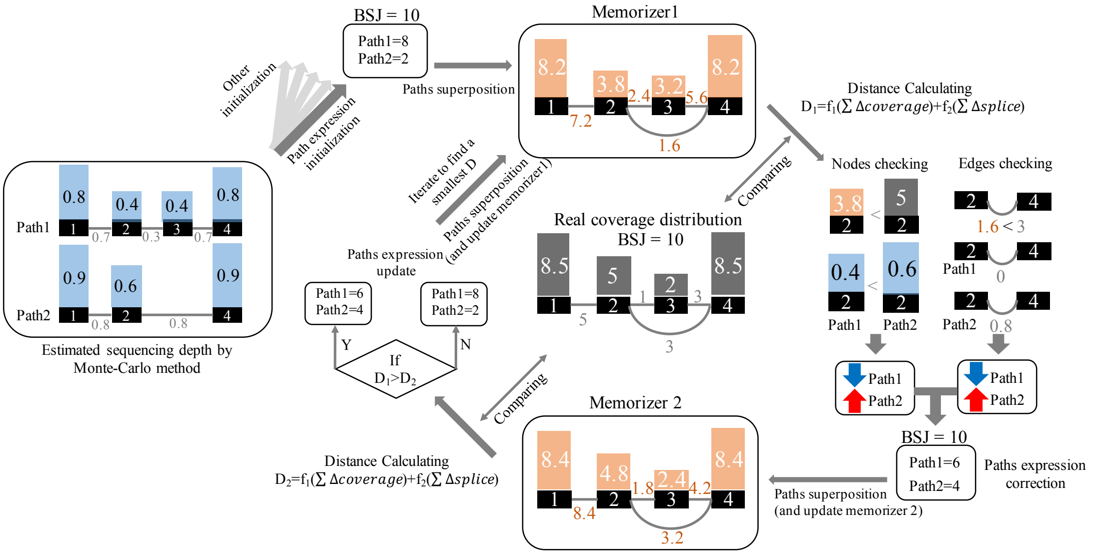

Figure S7. Workflow of approximate exhaustive search in the FSG algorithm. A Monte-Carlo method estimates the coverage and splice numbers on each path per BSJ read pair (left). Approximate exhaustive search starts by assigning a random putative abundance to each path, where the summed abundance for all paths should be equal to the total number of BSJ reads. Then, based on the assigned putative abundance and the distribution of simulated BSJ-reads, the putative abundances of nodes and edges on each path are computed. Based on the resulting abundance of nodes and edges, accumulated putative abundance of nodes and edges are calculated and the distance between putative and real abundance (inferred from mapped BSJ-reads) of nodes and edges is calculated and recorded. Next, the putative abundances of paths are adapted to real abundance of nodes and edges. Finally, this iteration process stops when the distance gets converged.

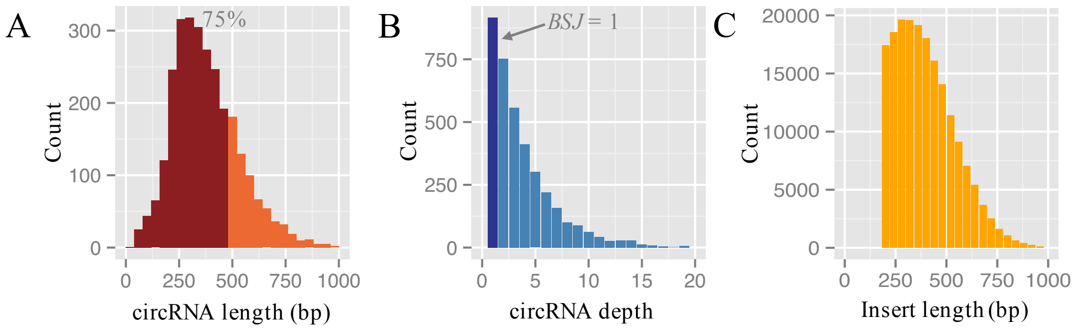

Figure S8. Characteristics of simulated data sets. (A) The length distribution of circRNAs in the Hela dataset (SRR3476958, SRR3476956 and SRR3479116), and this distribution is used in the simulated RNA-seq data set. Dark red bars represent circRNAs with length ≤ 480bp, which theoretically can be recovered by 250bp paired-end reads. (B) The distribution of BSJ read counts in the simulated RNA-seq data set with an average of 5X circRNA coverage. (C) The distribution of insert fragment length in the simulated data (μ=350, σ=200). Read pairs with insert length shorter than read length are removed.

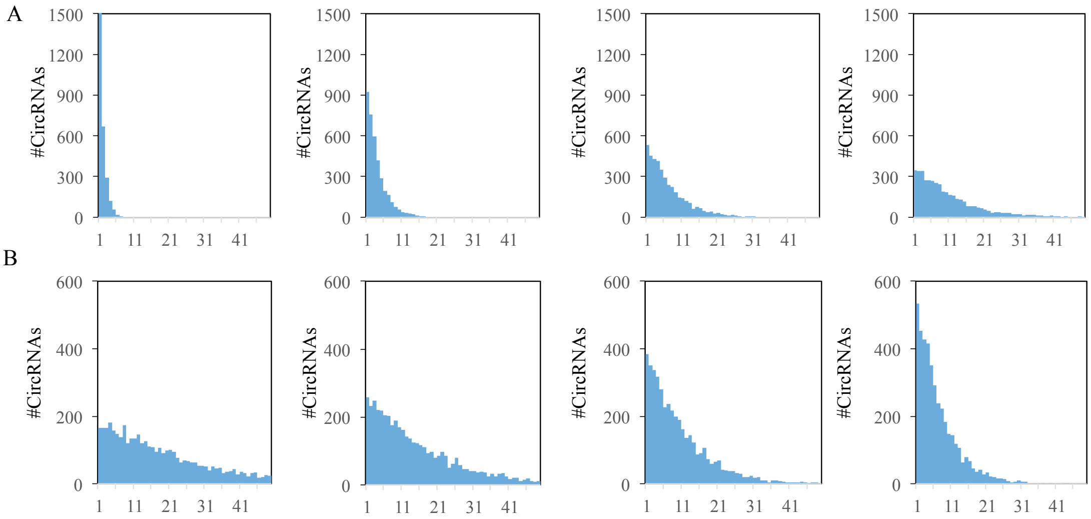

Figure S9. Abundance distributions of circRNAs in simulated data sets. These data sets share the same insert length distribution (normal distribution: μ=350, σ=200). X axis represents the expression level as measured by the number of BSJ reads. (A) Four simulated data sets with different circRNA sequencing depth (average 2X, 5X, 10X, 15X) and identical read length (PE200) (B) Four simulated data sets with different read length (PE75, PE100, PE150, PE200) and identical average abundance of circRNAs (10X).

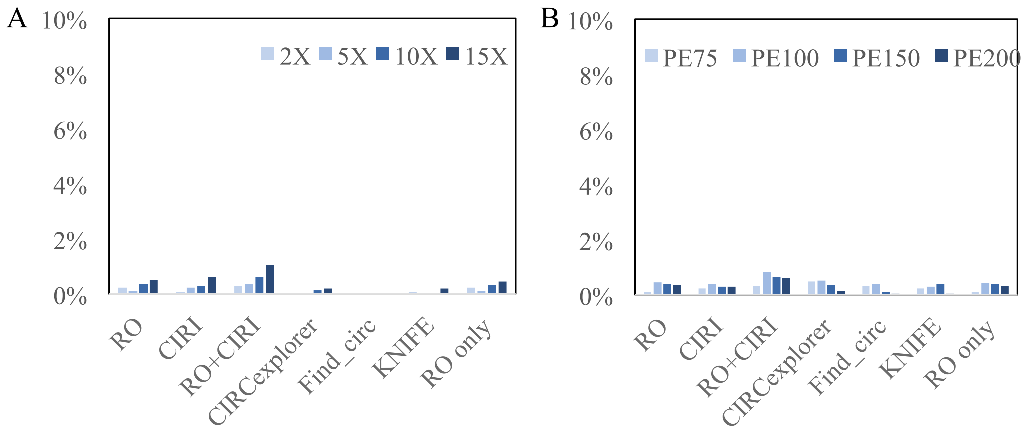

Figure S10. False discovery rate (FDR) of different circRNA detection tools on simulated data sets. 'CIRI+RO' represents for the combination of circRNAs found by CIRI and RO. 'RO only' represents the circRNAs that are specifically detected by the RO method. (A) FDR in the simulated data sets with different circRNA sequencing depth and FDR in the simulated data sets with different read length.

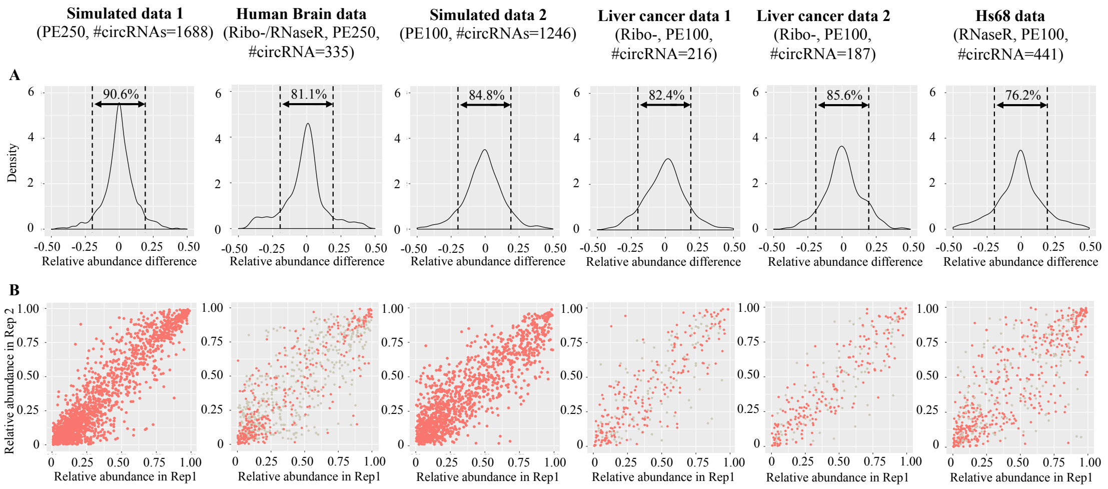

Figure S11. CircRNA quantification in four real RNA-seq data sets (human brain, liver cancer dataset1, liver cancer dataset2 and Hs68) with biological replicates and two simulated datasets. (A) Density plot of the relative abundance difference of highly expressed circRNA isoforms (#BSJ counts >= 30) between replicates. (B) Scatterplot of the relative abundance of circRNA isoforms between replicates. Red dots represent the highly expressed circRNA isoforms (#BSJ counts >=30), and gray dots represent the moderately expressed circRNA isoforms (#BSJ counts < 30 & >10).

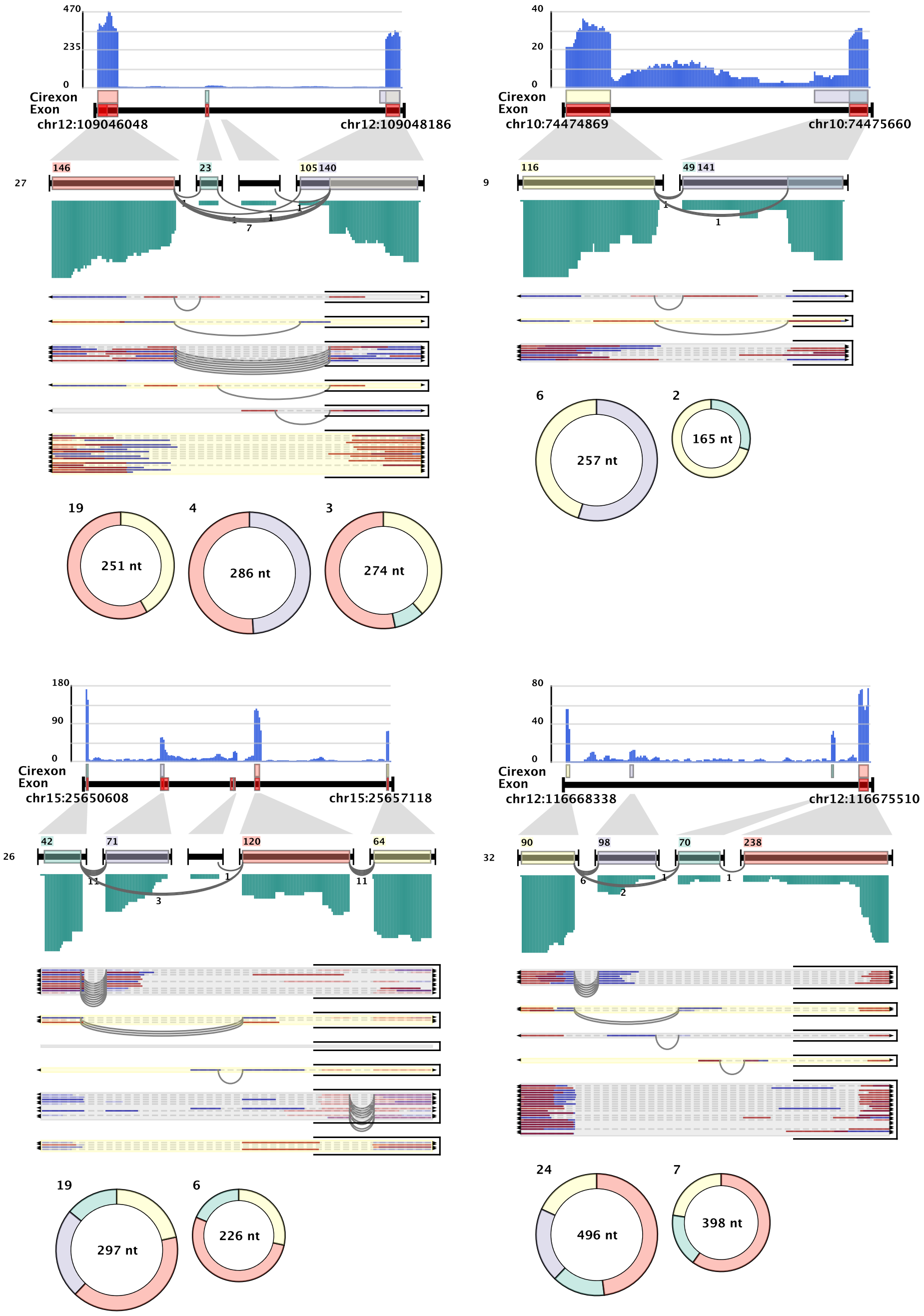

Figure S12. Verified circRNA structure and their predicted relative abundance in total RNA-seq data set with read length of 100 bp. X and Y axes denote the same as those in **Figure S2**.

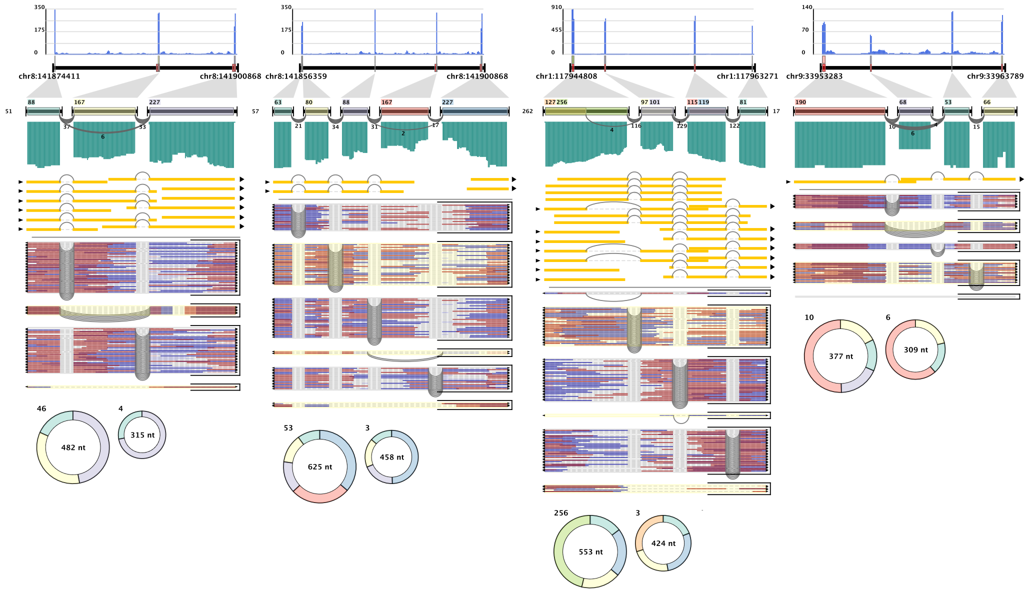

Figure S13. Verified circRNA structure and their predicted relative abundance in total RNA-seq data set with read length of 250 bp. X and Y axes denote the same as those in **Figure S2**.

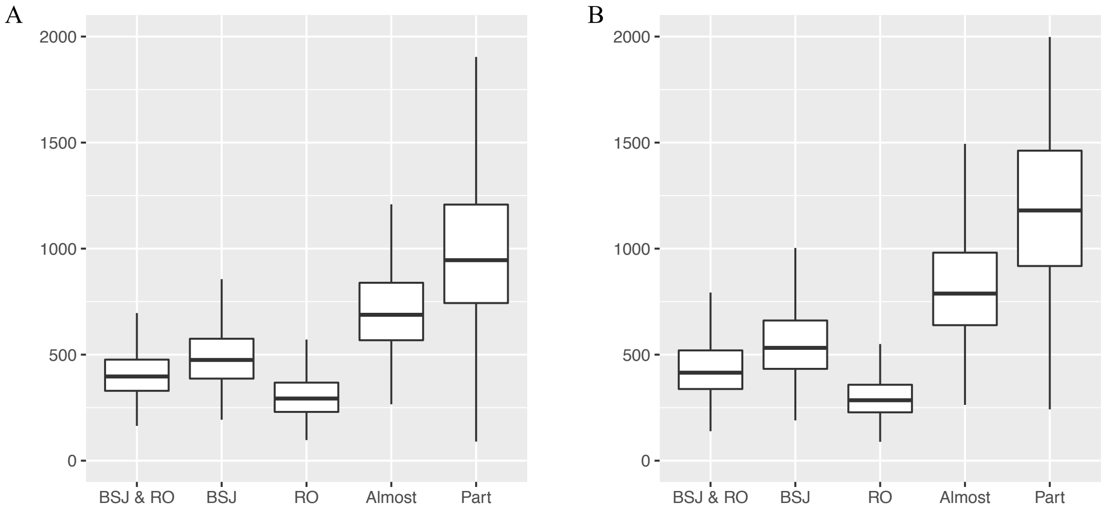

Figure S14. Length of circRNAs detected by CIRI-full in human HeLa cell line. (A) and human whole brain tissue (B). CircRNAs are reconstructed by RO reads (RO), BSJ reads (BSJ) and the combination of both BSJ and RO.

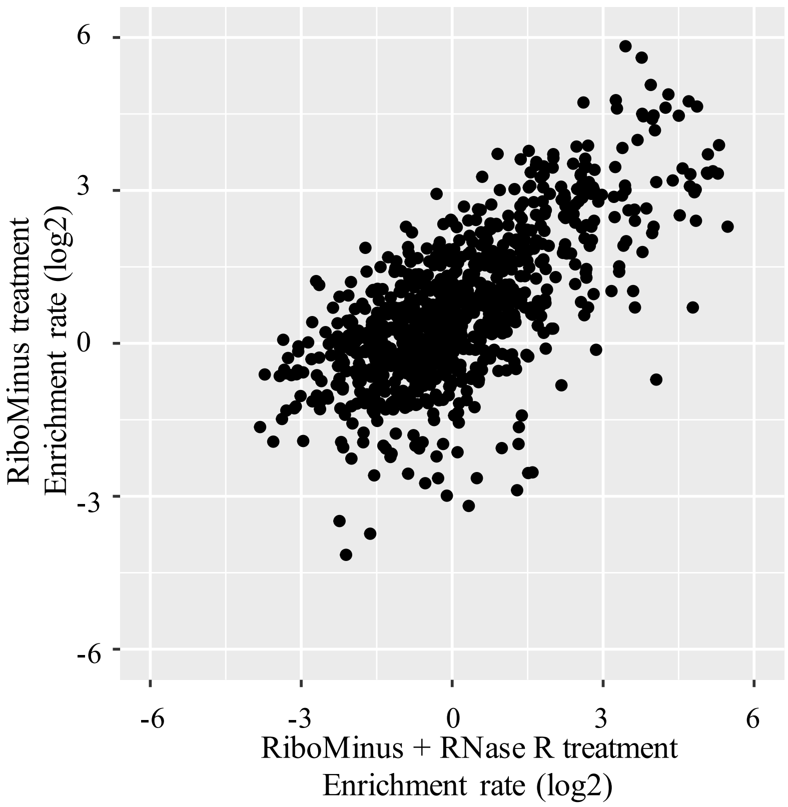

Figure S15. Cirexon enrichment rate in the RiboMinus treatment sample and RNase R+RiboMinus treatment sample as compared to the Poly (A) enrichment sample. Each dot represents the cirexon (uniquely found by RO method) enrichment rate in the RiboMinus treatment sample (y-axis) and RNase R + RiboMinus treatment sample (x-axis). Enrichment rate was calculated as (cirexon_average_coverage) / (Poly(A)+ cirexon_average_coverage). Coverage was normalized by data size.

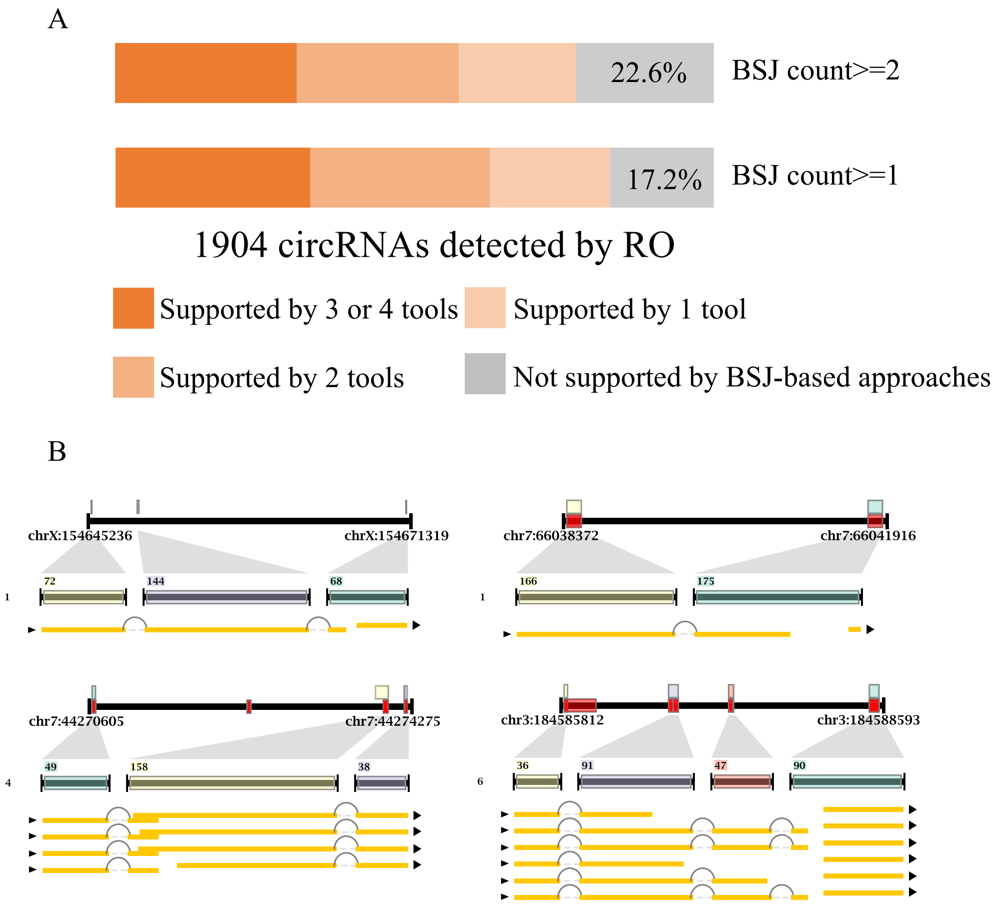

Figure S16. RO feature is reliable in detecting lowly expressed circRNAs. (A) Most of the RO-detected circRNAs could be confirmed by previous BSJ-based approaches, including CIRI2, CircExplorer2, DCC and Mapsplice2. (B) Four examples of lowly-expressed circRNAs that were only detected by the RO feature.

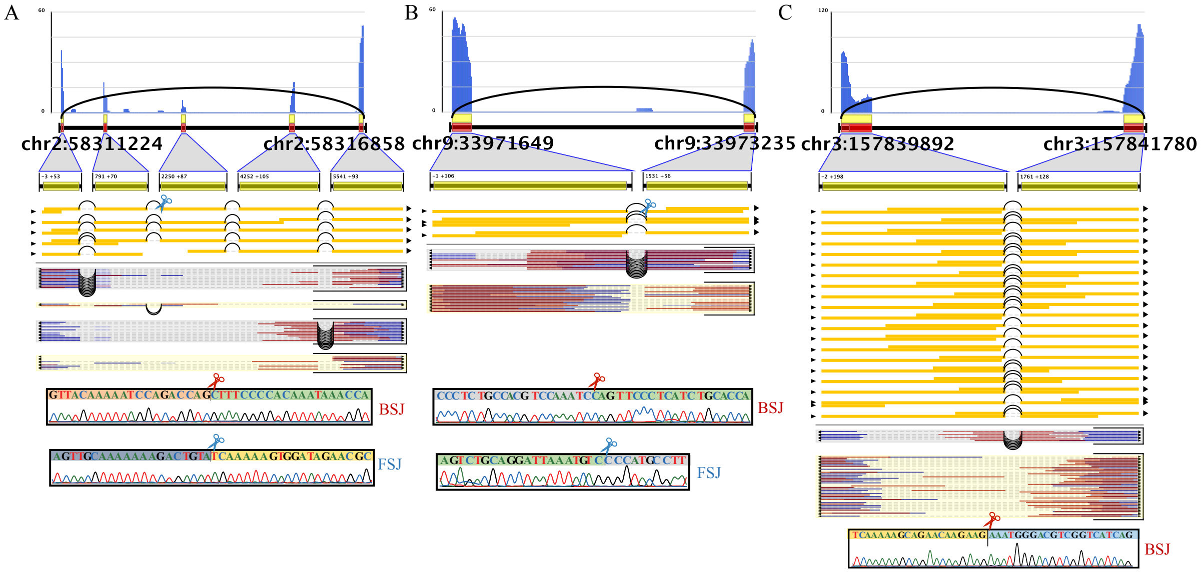

Figure S17. Experimental validations of the RO method on detecting highly-expressed circRNAs (# BSJ reads >= 30) in HeLa cell line. X and Y axes denote the same as those in **Figure S4**. Outward-facing primers are designed to confirm the BSJs of circRNAs detected by the RO method. PCR products are cloned and their sequences were determined by Sanger sequencing.

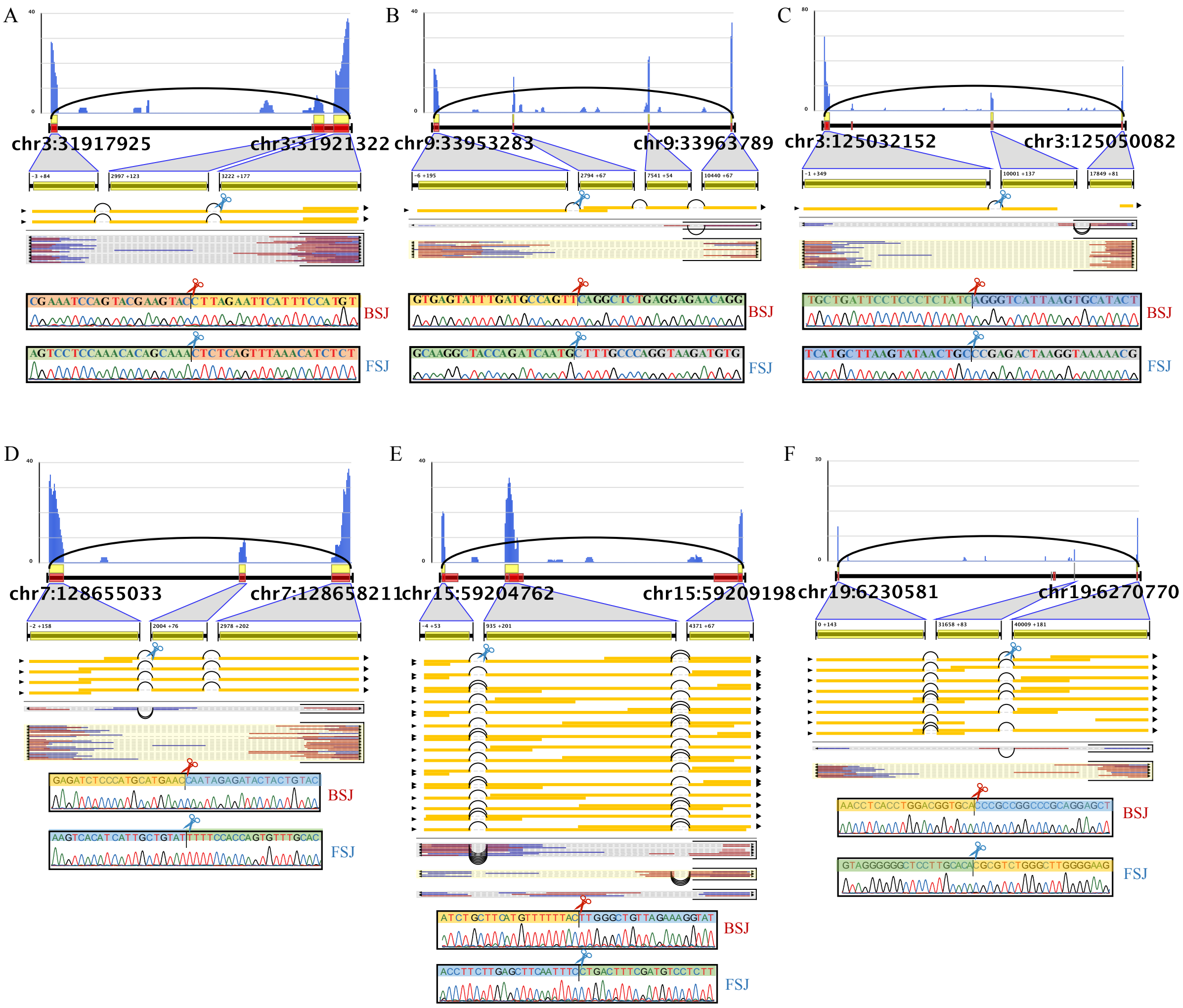

Figure S18. Experimental validations of the RO method on detecting moderately- expressed circRNAs (# BSJ reads >= 10 & < 30) in HeLa cell line. X and Y axes denote the same as those in **Figure S4**. Outward-facing primers are designed to confirm the BSJs of circRNAs detected by the RO method. PCR products are cloned and their sequences were determined by Sanger sequencing.

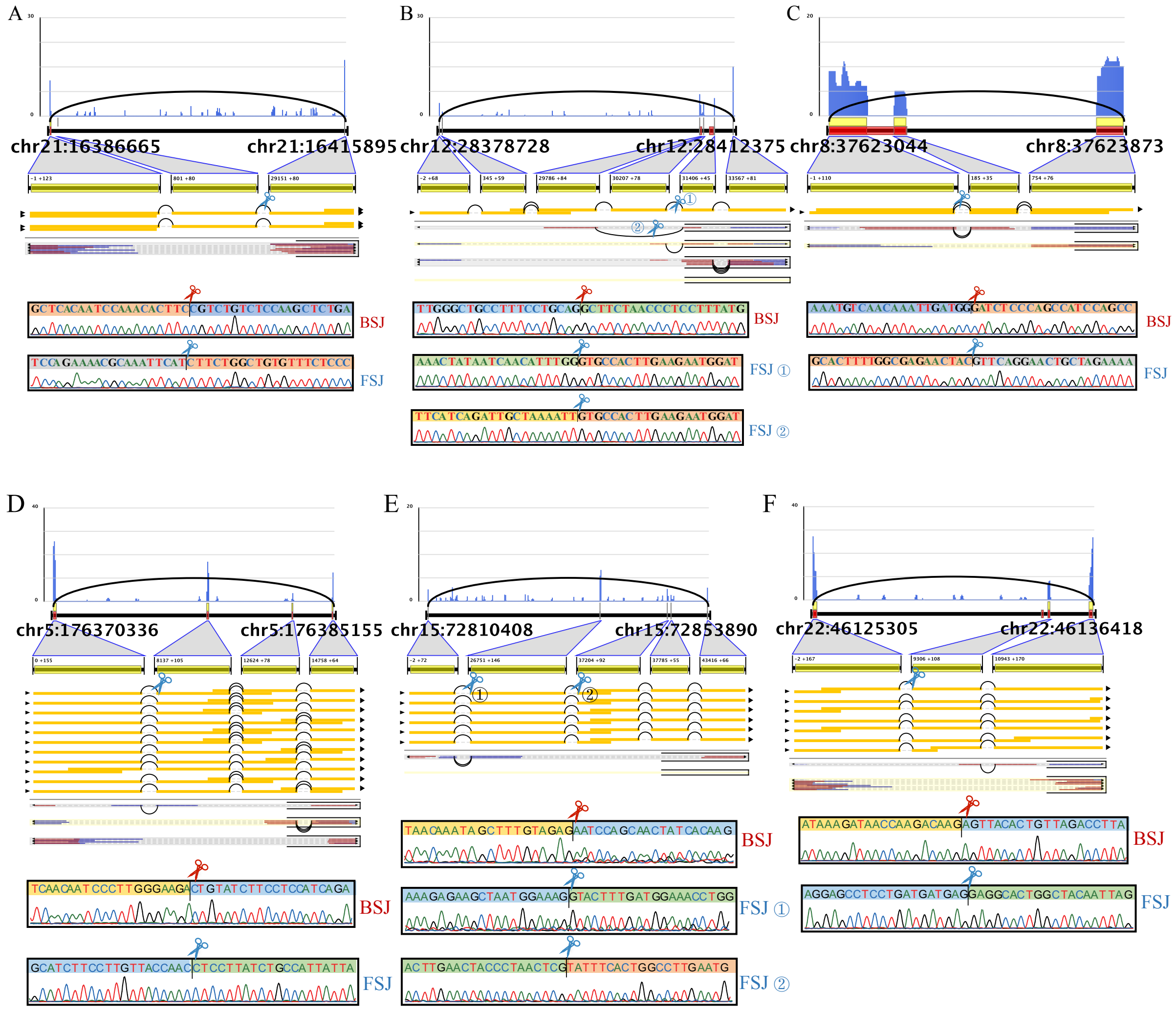

Figure S19. Experimental validations of the RO method on detecting weakly-expressed circRNAs (# BSJ reads < 10) in HeLa cell line. X and Y axes denote the same as those in **Figure S4**. Outward-facing primers are designed to confirm the BSJs of circRNAs detected by the RO method. PCR products are cloned and their sequences were determined by Sanger sequencing.

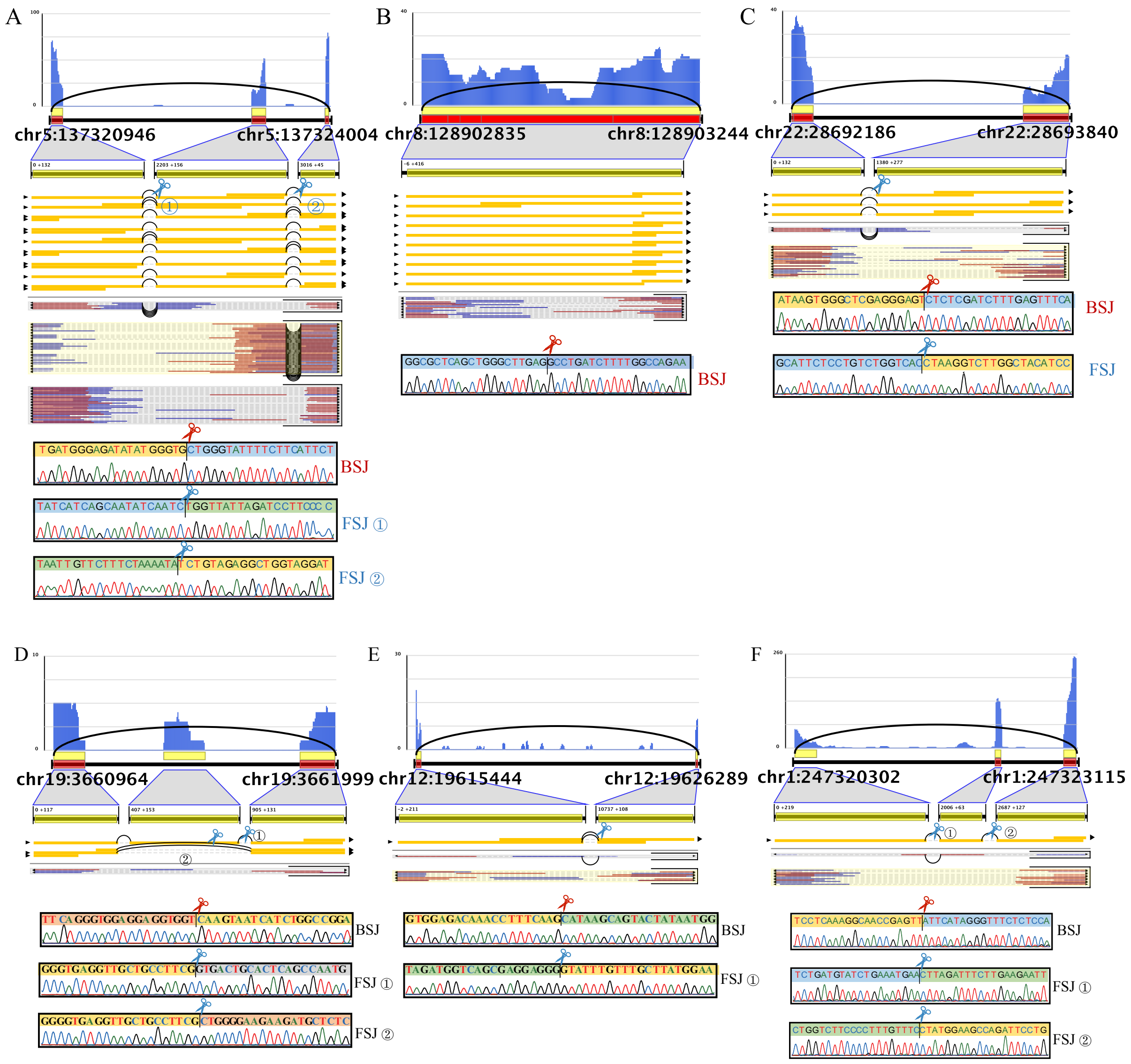

Figure S20. Experimental validations of the RO method on reconstructing full-length circRNAs in HeLa cell line. X and Y axes denote the same as those in **Figure S4**. Outward-facing primers are designed to confirm the BSJs and FSJs of circRNAs detected by the RO method. PCR products are cloned and their sequences were determined by Sanger sequencing.

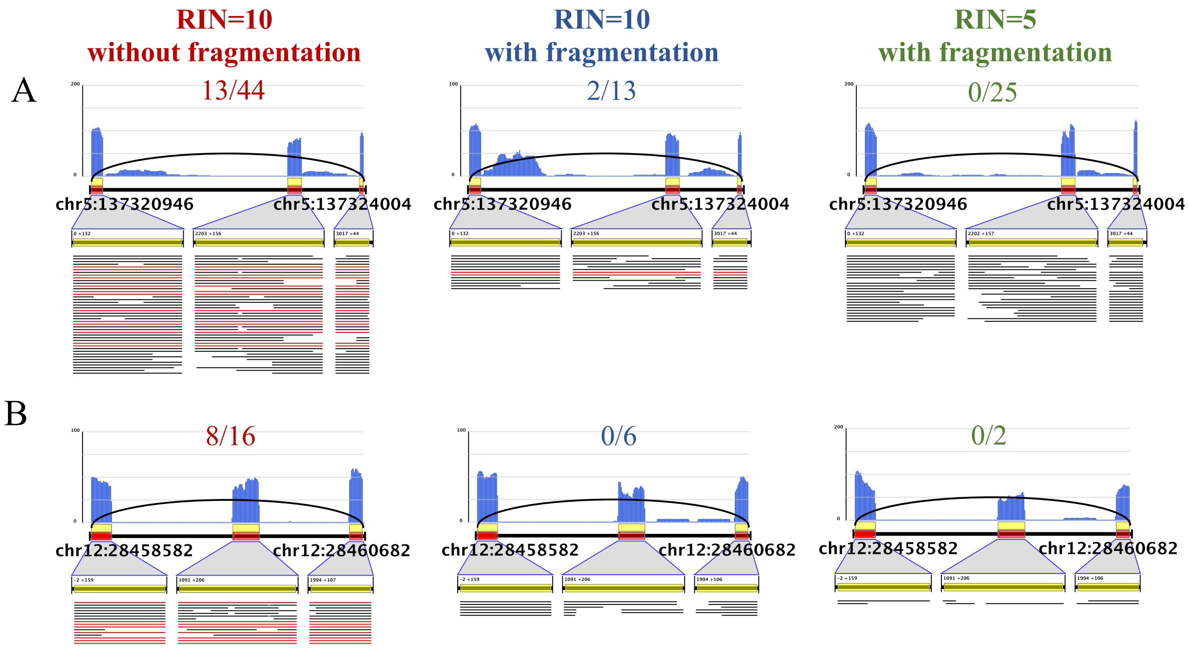

Figure S21. RNA degradation and fragmentation can reduce the abundance of RO reads. Two examples of circRNAs are illustrated to display the mapping of RO and BSJ reads in the three RiboMinus/RNase R treated HeLa cell RNA-seq data sets, RIN=10 & without fragmentation, RIN=10 with fragmentation, and RIN=5 with fragmentation. Red and black lines represent RO reads and BSJ reads, respectively. (A) CircRNA (chr5:127,320,946|137,324,004) with length of 329 bp. The number of RO/BSJ reads in the three data sets is 13/44, 2/13, 0/25, respectively. (B) CircRNA (chr12:28,458,582|28,460,682) with length of 469 bp. The number of RO/BSJ reads in the three data sets is 8/16, 0/6, 0/2, respectively.

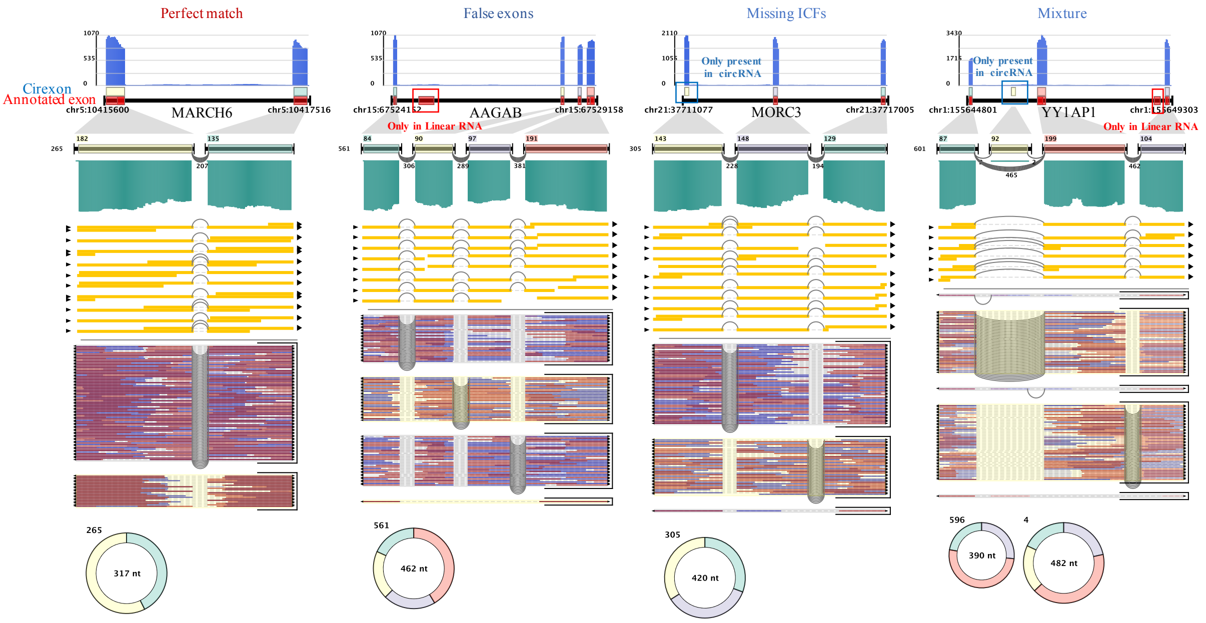

Figure S22. Four examples of circRNAs in Figure 3K. Annotated exons in GTF (Gencode v19) are represented by red boxes on the black axis. Colored boxes above the black axis represent predicted cirexons.

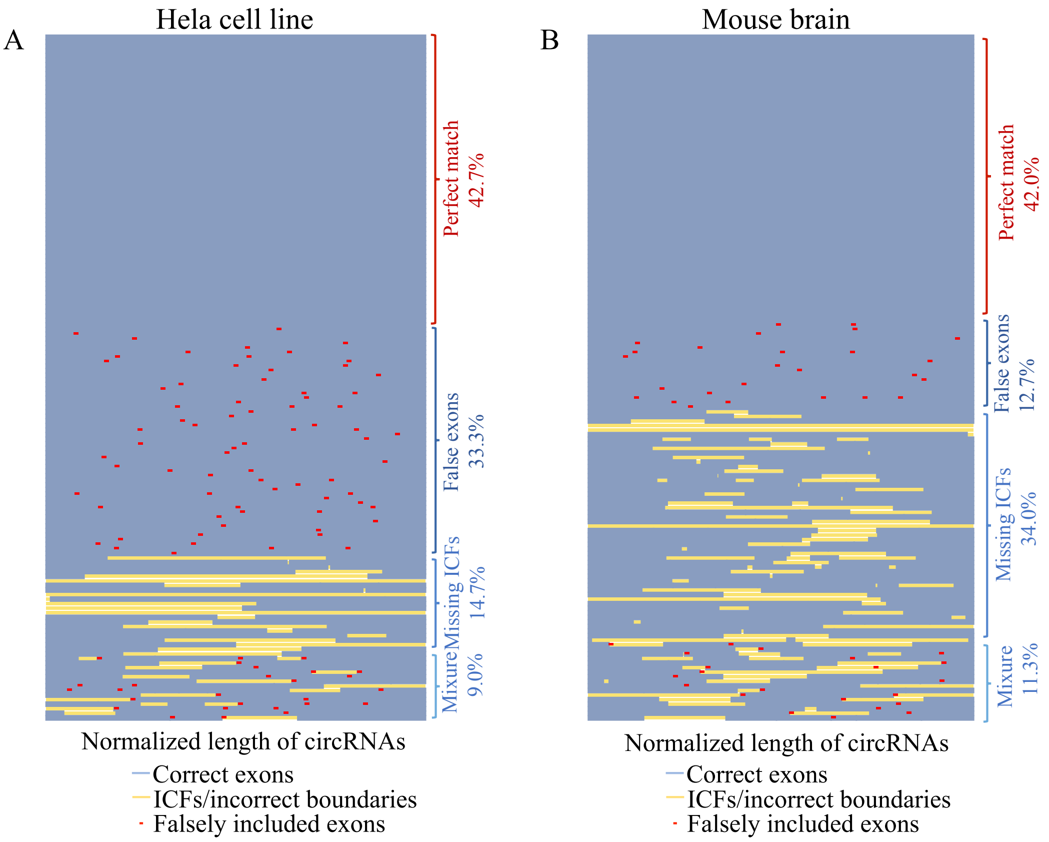

Figure S23. Comparison of full length circRNA structure and corresponding annotated exon regions in human HeLa cell line (A) and mouse brain tissue (B). For each data set, top 150 highly expressed circRNAs which are completely reconstructed are shown. Each line represents a circRNA with normalized length.

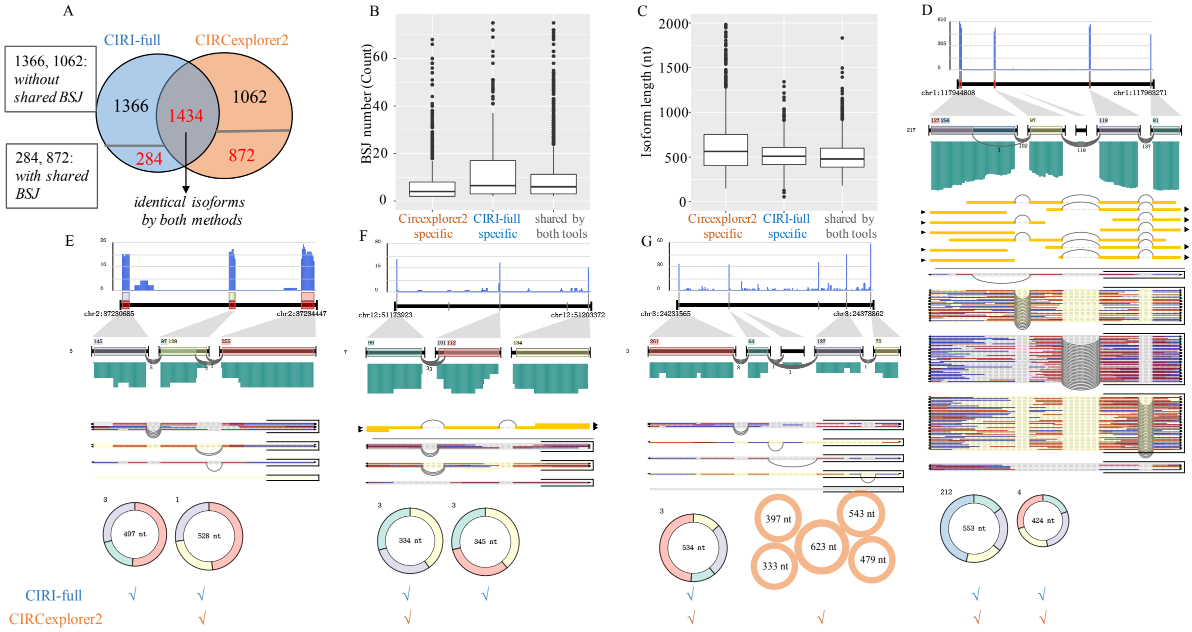

Figure S24. Performance comparison between CIRI-Full and CIRCexplorer2 on a human brain sub-dataset. This dataset was treated by RNase R & RiboMinus with 3 million read pairs. CircRNA with BSJ reads >=3 were retained. (A) Comparison of reconstructed circular isoforms between CIRI-full and CIRCexplorer2. Black number represents the number of isoforms that do not share BSJ with those predicted by the other tool. Red number represents the number of isoforms that share BSJ with the other tool but differ in their internal sequences. (B, C) Boxplot of BSJ level expression and isoform length of three types of isoforms shown in red color in Figure S23A. (D) An example of circRNA that all its isoforms were reconstructed by both tools. (E, F) Examples of isoforms only predicted by CIRI-full. (G) Example of isoforms only predicted by CIRCexplorer2.

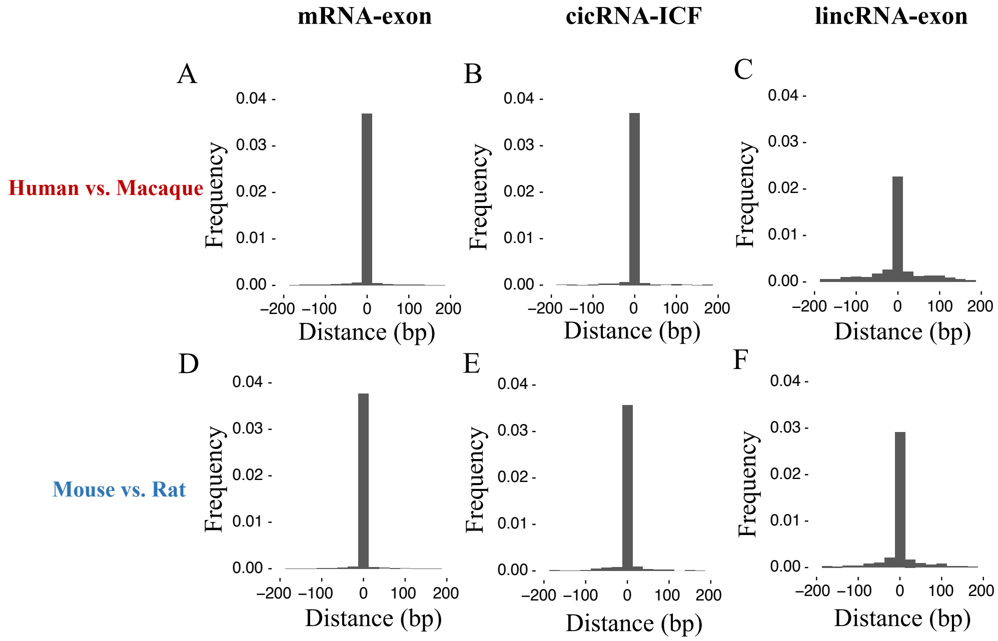

Figure S25. Boundary conservation of orthologous exons in mRNA, circRNA and lincRNA. For circRNA, only ICFs are taken into account. (A-C) Boundary conservation in orthologous genes between human and macaque. (D-F) Boundary conservation in orthologous genes between mouse and rat.

Table S1. RT-PCR and CIRI-full quantification results of 17 circRNA isoforms.

BSJ	Length	gene_name	expression	Y_axis	GAPDH_ mean	CT_Mean	PCR_result (2^(-△CT)*10000)	X_axis
chr12:109046048|109048186	274	CORO1C	3	11.1%	18.92444	32.07436	1.100220264	17.6%
chr12:109046048|109048186	286	CORO1C	5	18.5%	18.92444	30.85816	2.556185224	40.9%
chr12:109046048|109048186	251	CORO1C	19	70.4%	18.92444	30.83783	2.592461168	41.5%
chr10:74474869|74475660	165	MCU	5	55.6%	18.92444	31.79133	1.338690431	48.3%
chr10:74474869|74475660	257	MCU	4	44.4%	18.92444	31.69297	1.433142811	51.7%
chr12:116668338|116675510	398	MED13L	7	22.6%	18.92444	30.94609	2.405042485	32.7%
chr12:116668338|116675510	496	MED13L	24	77.4%	18.92444	29.90671	4.943190067	67.3%
chr15:25650608|25657118	226	UBE3A	6	23.1%	18.92444	30.75597	2.743813417	21.8%
chr15:25650608|25657118	297	UBE3A	19	76.7%	18.92444	28.91724	9.814483728	78.2%
chr8:141874411|141900868	482	PTK2	46	92.0%	17.02938	27.93915	5.197948642	98.2%
chr8:141874411|141900868	315	PTK2	4	8.0%	17.02938	33.68426	0.09691289	1.8%
chr8:141856359|141900868	625	PTK2	53	94.6%	17.02938	29.85471	1.377815181	90.2%
chr8:141856359|141900868	458	PTK2	3	5.4%	17.02938	33.05096	0.150322449	9.8%
chr1:117944808|117963271	553	MAN1A2	236	98.7%	17.02938	23.14939	143.7783238	100.0%
chr1:117944808|117963271	300	MAN1A2	3	1.3%	18.19529	36.79005	0.025259193	0.0%
chr9:33953283|33963789	377	UBAP2	10	62.5%	17.92756	30.13061	2.120879095	61.7%
chr9:33953283|33963789	309	UBAP2	6	37.5%	17.92756	30.82106	1.31422508	38.3%

- chr1:117944808|117963271
- chr10:74474869|74475660
- chr12:109046048|109048186
- chr12:116668338|116675510
- chr15:25650608|25657118
- chr8:141856359|141900868
- chr8:141874411|141900868
- chr9:33953283|33963789
